# Echocardiographic reference intervals with allometric scaling of 823 clinically healthy rhesus macaques (*Macaca mulatta*)

**DOI:** 10.1186/s12917-020-02578-y

**Published:** 2020-09-22

**Authors:** Yu Ueda, Laetitia M. M. Duler, Kami J. Elliot, Paul-Michael D. Sosa, Jeffrey A. Roberts, Joshua A. Stern

**Affiliations:** 1 Department of Clinical Sciences, College of Veterinary Medicine, North Carolina State University, Raleigh, NC 27606 USA; 2 Department of Medicine & Epidemiology, School of Veterinary Medicine, University of California-Davis, Davis, CA 95616-8732 USA; 3 California National Primate Research Center, University of California-Davis, Davis, CA 95616 USA

**Keywords:** Heart, Cardiac, Cardiovascular, Ultrasound, Non-human primate, Cardiomyopathy, Hypertrophic cardiomyopathy, Left ventricular hypertrophy

## Abstract

**Background:**

Echocardiography is commonly used for assessing cardiac structure and function in various species including non-human primates. A few previous studies reported normal echocardiographic reference intervals of clinically healthy rhesus macaques under sedation. However, these studies were under-powered, and the techniques were not standardized. In addition, body weight, age, and sex matched reference intervals should be established as echocardiographic measurements are commonly influenced by these variables. The purpose of this study was to establish reference intervals for complete echocardiographic parameters based on a large cohort of clinically healthy rhesus macaques with wide ranges of weight and age distributions using allometric scaling.

**Results:**

A total of 823 rhesus macaques (ages 6 months to 31 years old; body weights 1.4 to 22.6 kg) were enrolled. Of these rhesus macaques, 421 were males and 402 were females. They were assessed with a complete echocardiographic examination including structural and functional evaluation under sedation with ketamine hydrochloride. The reference intervals of the key echocardiographic parameters were indexed to weight, age, and sex by calculating the coefficients of the allometric eq. Y = aM^b^. On correlation matrix, body weight, age, sex, and heart rate were significantly correlated with various echocardiographic parameters and some of the parameters were strongly correlated with body weight and age. Multiple regression analysis revealed that heart rate and body weight statistically significantly predicted several echocardiographic parameters. Valve regurgitation including tricuspid, aortic, pulmonic, and mitral regurgitations without other cardiac structural and functional abnormalities are common in clinically healthy rhesus macaques under ketamine sedation.

**Conclusions:**

In this study, the reference intervals of echocardiographic parameters were established by performing complete echocardiographic examinations on a large number of clinical healthy rhesus macaques. In addition, allometric scaling was performed based on their weight, and further indexed to age and sex. These allometrically scaled reference intervals can be used to accurately evaluate echocardiographic data in rhesus macaques and diagnose structural and functional evidence of cardiac disease.

## Background

Echocardiography is a noninvasive imaging technique commonly used for assessing cardiac structure and function. It includes the measurement of cardiac chamber size and ventricular wall thickness, and an assessment of valve structure and function, systolic and diastolic function, and blood flow patterns in the heart. In clinical settings, findings from complete echocardiographic assessment are used as important information to determine medical and surgical treatment of cardiac diseases. In research settings, echocardiographic evaluation of subjects is inevitable to fully assess the cardiac condition for cardiovascular and non-cardiovascular research.

A few previous studies reported normal echocardiographic reference ranges of clinically healthy rhesus macaques under sedation [1, 2]. In addition, the authors of the present study reported echocardiographic reference intervals in geriatric rhesus macaques older than 18 years [3]. These studies reported that the reference intervals of various echocardiographic parameters in geriatric rhesus macaques are different from those in younger adult rhesus macaques. One of these studies also reported that diastolic dysfunction without other obvious cardiovascular abnormalities in geriatric rhesus macaques is common [3]. However, these previous studies did not have sufficient power due to their small sample sizes. In addition, these reference values were not indexed to body weight (BW). Weight matched reference intervals should be established due to the knowledge that various echocardiographic parameters are influenced by BW [4–6]. In addition, the reference intervals should be indexed to age and sex since some of variables are known to be influenced by these variables [7–9]. Previously, naturally occurring hypertrophic cardiomyopathy (HCM) and other cardiomyopathies have been reported in rhesus macaques and they could represent an important non-human primate model of human familial HCM [10–15]. Applying BW independent reference intervals to rhesus macaques may result in misdiagnosis of HCM as well as other cardiac diseases. The establishment of echocardiographic reference intervals indexed to these variables in a large population of rhesus macaques of various age and sex groups is fundamental to assessing their cardiovascular conditions and abnormalities in clinical and experimental settings. Knowing the normal reference values is of utmost importance to aid in the identification of the presence and severity of cardiovascular abnormalities.

The purpose of the present study was to establish reference intervals of key echocardiographic parameters based on a large cohort of clinically healthy rhesus macaques with wide ranges of weight and age distributions and establish these reference intervals with allometric scaling to BW. They are also indexed to other patient characteristics including the sex and age. This cohort of rhesus macaques represents the largest sample size for establishing echocardiographic parameters in the literature in any other veterinary patients.

## Results

A total of 823 rhesus macaques were enrolled in the present study. The average age (+/− standard deviation [SD]) and BW (+/−SD) were 7.8 (+/− 5.4) years and 8.49 (+/− 3.52) kg, respectively. The mean heart rate (HR) during the echocardiographic studies was 134 (+/− 24.6) bpm. Of 823 rhesus macaques, 421 rhesus macaques were male and 402 were female. The mean age (+/− SD) in males and females were 7.9 (+/− 5.4) years and 7.8 (+/− 5.4) years, respectively. The mean BW in males and females were 8.46 (+/− 3.54) kg and 8.46 (+/− 3.53) kg, respectively. The mean HR during the studies was 133 (+/− 24.6) bpm in males and 134 (+/− 24.6 bpm) in females.

None of the rhesus macaques had evidence of significant cardiac structural abnormalities or moderate to severe valve regurgitation. None of the rhesus macaques had systolic dysfunction determined based on left ventricular fractional shortening (LV FS) and left ventricular ejection fraction (LV EF). However, some of the older animals in the study group had evidence of diastolic dysfunction without other structural and/or functional abnormalities, which could be attributed to the age-related findings based on previous studies in rhesus macaques and other species [3]. Of the 823 rhesus macaques, 303 had tricuspid (36.8%), 119 had aortic (14.5%), 107 (13.0%) had pulmonic, and 92 had mitral (11.2%) valve regurgitation. All of these regurgitations were graded as trace or mild and they were not associated with chamber enlargement and/or cardiac dysfunction.

Intra-observer measurement variability for all echocardiographic variables measured in 10 rhesus macaques was between 0 and 6.5%, and interobserver measurement variability was between 0 and 10.7%.

Mean, SD, range, and reference interval with 90% confidence intervals of upper and lower limits for each two-dimensional (2D) and M-mode echocardiographic measurement are listed in Table 1. Continuous and pulsed-wave Doppler-derived parameters are listed in Table 2. On correlation matrix, WT, age, sex, HR, and blood pressure (BP) were significantly correlated with various echocardiographic parameters (Supplemental Table 1) with some parameters demonstrating strong correlation to BW and/or age. No significant correlation was found between the age and HR of rhesus macaques enrolled in the study (*r* = − 0.011, *P* = 0.8).

**Table 1:** 2-dimensional and M-mode measurements in rhesus macaques

				Reference Intervals	
Echo parameters	Mean	SD	Range	Lower limit	Upper limit
LAd (sa) (cm)	1.81	0.29	1.01–2.98	1.12	2.64
Ao (sa) (cm)	1.29	0.22	0.71–2.15	0.79	1.96
LA/Ao (sa)	1.41	0.19	0.96–2.14	1.02	2.03
LAd (la) (cm)	2	0.32	0.39–3.68	1.01	2.9
Ao (la) (cm)	1.01	0.18	0.32–1.67	0.61	1.59
LA/Ao (la)	2.02	0.28	1.12–2.86	1.17	2.76
IVSd (2D) (cm)	0.54	0.1	0.3–0.91	3.19	8.72
LVPWd (2D) (cm)	5.97	1.01	2.17–9.7	3.5	9.4
IVSd (M-mode) (cm)	0.51	0.1	0.25–0.94	0.27	0.86
LVd (M-mode) (cm)	2.18	0.35	1.02–3.99	1.32	3.2
LVPWd (M-mode) (cm)	0.56	0.11	0.29–1.03	0.33	0.89
IVSs (M-mode) (cm)	0.75	0.16	0.38–1.42	0.4	1.22
LVs (M-mode) (cm)	1.33	0.29	0.34–3.1	0.68	2.31
LVPWs (M-mode) (cm)	0.84	0.18	0.41–1.95	0.47	1.42
EPSS (cm)	0.27	0.1	0.036–0.74	0.08	0.64
TAPSE (cm)	1.17	0.25	0.5–1.9	0.6	1.8

*IVSd* Interventricular septal thickness in diastole, *IVSs* Interventricular septal thickness in systole, *LVDs* Left ventricular internal diameter in systole, *LVPWd* Left ventricular posterior wall thickness in diastole, *LVPWs* Left ventricular posterior wall thickness in systole, *LA (la)* Left atrial diameter in diastole from long-axis view, *LA (sa)* Left atrial diameter in diastole from short-axis view, *TAPSE* Tricuspid annular plane systolic excursion

**Table 2:** Doppler-derived echocardiographic parameters in geriatric rhesus macaques

				Reference Intervals	
Echo parameters	Mean	SD	Range	Lower limit	Upper limit
Ao Vmax (m/sec)	1.12	0.21	0.53–1.77	0.6	1.64
A wave (m/sec)	0.57	0.14	0.19–1.0	0.3	0.84
E wave (m/sec)	0.85	0.18	0.45–1.5	0.5	1.2
E/A	1.54	0.40	0.91–3.67	0.75	2.33
LV IVRT (sec)	55.1	9.09	30–85	37.3	72.9
E: LV IVRT	0.02	0.0059	0.01–0.04	0.004	0.027
E’ wave medial (m/sec)	0.071	0.014	0.037–0.12	0.042	0.099
A’ wave medial (m/sec)	0.052	0.021	0.028–0.52	0.012	0.093
E’/A’ medial	1.41	0.32	0.9–2.74	0.78	2.0
E/E’ medial	12.4	3.0	0.62–23.8	6.52	18.28
E’ wave lateral (m/sec)	0.1	0.022	0.045–0.16	0.06	0.15
A’ wave lateral (m/sec)	0.06	0.016	0.028–0.06	0.029	0.092
E’/A’ lateral	1.77	0.48	0.96–3.87	0.83	2.71
E/E’ lateral	8.7	2.24	4.49–19.7	4.3	13.1
PV Vmax (m/s)	1.021	0.17	0.61–1.87	0.67	1.65
PV ET (sec)	216.98	25.71	150–300	133.97	288.52
PV AT (sec)	77.59	15.86	21–132	29.47	123.02
PV AT/ET	0.36	0.066	0.2–0.57	0.17	0.58
RV S′ Vmax (m/s)	0.096	0.024	0.04–0.17	0.04	0.17

A wave, passive filling velocity; A’ wave, late diastolic mitral annulus motion; E wave, atrial contraction velocity; E’ wave, early diastolic mitral annulus motion: LV IVRT; left ventricular isovolumic relaxation time; PVAT, pulmonary valve acceleration time; PVET, pulmonary valve ejection time; Ao Vmax, peak velocity for aortic flow; PV Vmax, peak velocity for pulmonary flow; RV S′ Vmax, tricuspid peak systolic annular velocity

Multiple regression analysis was done to predict various echocardiographic parameters from weight, age, sex and HR. Among these animal characteristics, weight and HR statistically significantly predicted various echocardiographic parameters (Supplement Table 2). Regression analysis using logarithmically transformed echocardiographic parameters including the proportionality constant (a) and allometric scaling exponents (b) are reported in Table 3. BW-based means and 95% prediction intervals of 2D and M-mode echocardiographic parameters derived from allometric scaling parameters in 823 clinically healthy rhesus macaques are listed in Table 4. The analysis was repeated after the animals were divided based on their sex (Supplement Table 3 and 4). The rhesus macaques were also sub-grouped into 5 months – 4 years old group, 5–9, 10–14, and ≥ 15 years old groups, and allometric scaling was performed within each age group (Supplement Table 5, 6, 7 and 8).

**Table 3:** Results of linear regression analysis of logarithmically transformed echocardiographic variables and body weight including the proportionality constants (a) and allometric scaling exponents (b) from clinically healthy rhesus macaques

Echocardiographic parameters	Proportionality constant (a)	SE of Y estimate	Scaling exponent (b)	SE of b	R^2^
LA (sa)	0.022	0.04998	0.258	0.00920	0.49
Ao (sa)	−0.148	0.04885	0.286	0.00899	0.56
LA (la)	0.072	0.05847	0.254	0.01069	0.41
Ao (la)	−0.278	0.05063	0.310	0.00926	0.58
IVSd (2D)	0.505	0.06733	0.256	0.01340	0.35
LVPWd (2D)	0.548	0.0565	0.256	0.01124	0.43
IVSd (M-mode)	−0.512	0.07535	0.241	0.01383	0.27
LVDd (M-mode)	0.101	0.04955	0.262	0.00909	0.51
LVPWd (M-mode)	−0.517	0.06389	0.292	0.01172	0.43
IVSs (M-mode)	−0.364	0.0758	0.260	0.01391	0.3
LVDs (M-mode)	−0.948	0.08478	0.236	0.01556	0.22
LVPWs (M-mode)	−0.366	0.06537	0.317	0.01200	0.46

*IVSd* Interventricular septal thickness in diastole, *IVSs* Interventricular septal thickness in systole, *LVDs* Left ventricular internal diameter in systole, *LVPWd* Left ventricular posterior wall thickness in diastole, *LVPWs* Left ventricular posterior wall thickness in systole, *LA (la)* Left atrial diameter in diastole from long-axis view, *LA (sa)* Left atrial diameter in diastole from short-axis view, *TAPSE* Tricuspid annular plane systolic excursion

**Table 4:** Body weight-based means and 95% prediction intervals of 2D and M-mode echocardiographic parameters derived from allometric scaling parameters in 823 clinically healthy rhesus macaques

Echo parameters	Body weight (kg)											
	1	2	4	6	8	10	12	14	16	18	20	22
LA (sa)	1.1	1.3	1.5	1.7	1.8	1.9	2	2.1	2.2	2.2	2.3	2.3
	0.87–1.27	1.04–1.52	1.24–1.82	1.38–2.02	1.49–2.18	1.58–2.3	1.65–2.42	1.72–2.51	1.78–2.6	1.83–2.68	1.88–2.76	1.93–2.82
Ao (sa)	0.7	0.9	1.1	1.2	1.3	1.4	1.4	1.5	1.6	1.6	1.7	1.7
	0.57–0.89	0.69–1.08	0.85–1.32	0.95–1.48	1.03–1.61	1.1–1.71	1.16–1.81	1.21–1.89	1.26–1.96	1.3–2.03	1.34–2.09	1.38–2.15
LA (la)	1.2	1.4	1.7	1.9	2	2.1	2.2	2.3	2.4	2.5	2.5	2.6
	0.97–1.43	1.16–1.7	1.38–2.03	1.53–2.25	1.65–2.42	1.74–2.56	1.83–2.68	1.9–2.79	1.97–2.89	2.02–2.97	2.08–3.06	2.13–3.13
Ao (la)	0.5	0.7	0.8	0.9	1	1.1	1.1	1.2	1.2	1.3	1.3	1.4
	0.43–0.64	0.54–0.79	0.37–0.98	0.76–1.11	0.83–1.22	0.89–1.31	0.94–1.38	0.99–1.45	1.03–1.51	1.07–1.57	1.1–1.62	1.14–1.67
IVSd (2D)	0.32	0.38	0.46	0.51	0.54	0.58	0.6	0.63	0.65	0.67	0.69	0.71
	0.25–0.41	0.3–0.49	0.35–0.59	0.39–0.65	0.42–0.7	0.45–0.75	0.47–0.78	0.49–0.81	0.5–0.84	0.52–0.87	0.53–0.89	0.55–0.91
LVPWd (2D)	0.35	0.42	0.5	0.56	0.6	0.64	0.67	0.69	0.72	0.74	0.76	0.78
	0.29–0.44	0.34–0.52	0.41–0.62	0.45–0.69	0.49–0.75	0.41–0.79	0.54–0.83	0.56–0.86	0.58–0.89	0.6–0.92	0.61–0.94	0.63–0.97
IVSd (M-mode)	0.3	0.4	0.4	0.5	0.5	0.5	0.6	0.6	0.6	0.6	0.6	0.6
	0.23–0.41	0.27–0.48	0.32–0.57	0.36–0.63	0.38–0.68	0.4–0.71	0.42–0.75	0.44–0.77	0.45–0.8	0.46–0.82	0.48–0.84	0.49–0.86
LVDd (M-mode)	1.3	1.5	1.8	2	2.2	2.3	2.4	2.5	2.6	2.7	2.8	2.8
	1.04–1.52	1.25–1.83	1.5–2.19	1.67–2.43	1.8–2.62	1.91–2.78	2–2.92	2.09–3.04	2.16–3.15	2.23–3.25	2.29–3.34	2.35–3.42
LVPWd (M-mode)	0.3	0.4	0.5	0.5	0.6	0.6	0.6	0.7	0.7	0.7	0.7	0.7
	0.24–0.39	0.29–0.47	0.36–0.58	0.40–0.65	0.44–0.71	0.47–0.76	0.49–0.8	0.52–0.84	0.54–0.87	0.55–0.9	0.57–0.93	0.59–0.96
IVSs (M-mode)	0.6	0.7	0.8	0.9	0.9	1	1.1	1.1	1.1	1.2	1.2	1.2
	0.32–0.58	0.39–0.69	0.47–0.83	0.52–0.92	0.56–0.99	0.59–1.05	0.62–1.1	0.65–1.15	0.67–1.19	0.69–1.23	0.71–1.26	0.73–1.29
LVDs (M-mode)	0.6	0.6	0.8	0.8	0.9	0.9	1	1	1.1	1.1	1.1	1.1
	0.58–1.11	0.69–1.31	0.81–1.54	0.89–1.69	0.95–1.81	1–1.91	1.05–1.99	1.09–2.07	1.12–2.13	1.15–2.19	1.18–2.25	1.21–2.3
LVPWs (M-mode)	0.6	0.7	0.9	1	1.1	1.1	1.2	1.3	1.3	1.4	1.4	1.5
	0.34–0.55	0.42–0.69	0.52–0.86	0.59–0.98	0.65–1.07	0.70–1.15	0.74–1.22	0.78–1.28	0.81–1.33	0.84–1.38	0.87–1.43	0.9–1.47

*IVSd* Interventricular septal thickness in diastole, *IVSs* Interventricular septal thickness in systole, *LVDs* Left ventricular internal diameter in systole, *LVPWd* Left ventricular posterior wall thickness in diastole, *LVPWs* Left ventricular posterior wall thickness in systole, *LA (la)* Left atrial diameter in diastole from long-axis view, *LA (sa)* Left atrial diameter in diastole from short-axis view, *TAPSE* Tricuspid annular plane systolic excursion

## Discussion

In this study, robust reference intervals for cardiac structure as well as left and right ventricular systolic and diastolic function of rhesus macaques were established. This study is highlighted by a large sample size with a broad range of age and weight as well as evenly distributed sexes. This is important because the statistical method for generating reference intervals and its accuracy with lower confidence intervals of reference limits are dependent on the total sample size and its distribution [16]. Another positive feature of the present study is that all echocardiographic images were obtained or assessed by an American College of Veterinary Internal Medicine board-certified veterinary cardiologist (JS) using standardized methodology. This is also important because echocardiography is highly operator dependent and echocardiographic images obtained with poor techniques might result in misdiagnosing cardiac conditions [17]. Although a few previous studies reported the reference intervals for various echocardiographic parameters in rhesus macaques, the sample numbers were relatively small and techniques to obtain echocardiographic images were not standardized [1–3]. Furthermore, allometric scaling of echocardiographic evaluation based on BW was not performed in any of the previous studies. The present study provides more precise reference intervals based on BW as well as age and sex. These updated reference intervals will be used to assess cardiovascular condition as well as aid in diagnosing cardiac diseases such as HCM in rhesus macaques.

The results of the present study in 823 clinically healthy rhesus macaques demonstrated that BW, age, HR and sex have a significant effect on various 2D and M-mode echocardiographic parameters. Among all these variables, as reported in the previous studies, BW has the most significant impact on the echocardiographic measurement of left atrial and aortic diameter as well as left ventricular wall thickness during systole and diastole and performing allometric scaling eliminated the effect of BW on these measurements [18–22]. Therefore, using BW-based 95% prediction intervals based on allometric scaling is recommended when evaluating individual rhesus macaques. Since the age of rhesus macaques also has a significant impact on various echocardiographic parameters, using the 95% prediction intervals for the parameters in each age group (5 months - 4 years old, 5–9, 10–14, ≥15 years old) should be considered, in particular when the left ventricular wall thickness is close to the upper end of the weight-based 95% prediction intervals (Supplement Table 5, 6, 7 and 8). Although significant correlations were identified between various echocardiographic parameters and HR as well as sex, the correlations were all weak and less likely to have significant on echocardiographic assessment. However, due to the fact that the sex often has a significant impact on the variabilities of cardiac structure and function as well as progression of various cardiac diseases such as HCM in other species, BW-based 95% prediction intervals by allometric scaling were provided for each sex group for various echocardiographic variables (Supplement Tables 3 and 4) [7–9].

Mild valve regurgitation especially at the tricuspid and aortic valves were common in this population of rhesus macaques, particularly in older patients. This is consistent with the findings of a previous study [3]. Mild forms of valve regurgitation could be associated with sedation but also could be due to nonclinical mild valvular degeneration or other clinically insignificant congenital or acquired valve diseases [23, 24]. Valve regurgitation could also be associated with pulmonary or systemic hypertension, however these findings were ruled out in our study based upon systolic blood pressure measurements performed in a portion (*n* = 182) of animals and lack of quantitative or qualitative structural heart changes expected with these conditions [24, 25]. This represents one minor limitation of this study since systolic BP measurements were not performed in all animals as it was added to the routine echocardiographic protocol at a later date during data collection. Thus, although unlikely, mild systemic hypertension could not be ruled out as a sole or partial cause of trace or mild aortic or mitral valve regurgitation in these animals [26]. Ultimately, the authors content that the absence of other cardiac structural and functional abnormalities as well as the absence of clinical signs in association with systemic hypertension rules out significant systemic hypertension as a cause of valve regurgitation in these cases [27]. No further investigation into the incidence of trace or mild valve regurgitation was performed in this study but could offer important value for future translational research.

In this study, geriatric rhesus macaques ≥18 years old with diastolic dysfunction were included for establishment of reference intervals for various echocardiographic parameters as long as the rhesus macaques did not have any concurrent structural or functional abnormalities. However, the echocardiographic measurements from these geriatric animals directly affected by the presence of diastolic dysfunction were excluded. Although diagnosing diastolic dysfunction was not the main objective of this study, it should be noted that more robust measures of diastolic dysfunction could be considered for future investigations such as flow propagation velocity. Measurement of flow propagation velocity has been validated in various species including humans, dogs and cats for diagnosis of diastolic dysfunction. Although it may be considered one of the gold standard measures for assessing preload-independent left ventricular relaxation in people, it varies significantly depending on heart rate, age, and percentage of segmental wall dyssynergy in dogs [28–30]. In addition to decreased E/A and E’/A’, elevated E/A and E/E’ have also been reported to be associated with the presence of diastolic dysfunction or mitral valve regurgitation in humans [31, 32]. Although cut-offs for elevation of these values has never been established in the rhesus macaque, some animals enrolled in this study had elevated E/A and E/E’ in the absence of valve regurgitation when compared to the reported human reference ranges. In the absence of diastolic dysfunction or valve regurgitation, these parameters can also be elevated due to hyperdynamic filling of the left ventricle during the early phase of diastole. This supranormal filling of the left ventricle has been reported in young and physically active people [33–35], and it stands to reason that this would be a reasonable explanation in our population of rhesus macaques. Further studies remain necessary to determine the most accurate way to diagnose diastolic dysfunction rhesus macaques.

The use of experimental animal models is imperative to advance our understandings of the pathogenesis and pathophysiology of human diseases [36]. It is also essential to study efficacy and safety of pharmaceutical compounds. Murine models of various diseases are the most commonly used as experimental animal models for studying human diseases including cardiovascular diseases. However, these animal models possess various hurdles which make the direct translational approach difficult [37]. These challenges include their small body size and their different cardiovascular physiology and kinetics. Large animal models including dogs, cats and pigs are also used for studying various diseases, but they still possess inherent physiological, biochemical and genetic differences from human beings [36, 38, 39]. Non-human primate models of cardiovascular diseases have the greatest advantages for translational research because of their physiological, biological, metabolic and genetic similarities to humans and thus they have played a key role to advance our understanding of diseases and clinical medicine [36, 40, 41]. Among them, rhesus macaques (*Macaca mulatta*) have been extensively used as a non-human primate model of human diseases. The reference intervals established by this study will therefore be important to utilize when researchers conduct future translational research in rhesus macaques for cardiovascular and non-cardiovascular diseases.

Accurate reference intervals are essential to diagnose various cardiac diseases impacting the health of rhesus macaques in research and clinical settings. For example, HCM has been reported at the California National Primate Center (CNPRC) and this naturally occurring cardiomyopathy is related to sudden cardiac death in this facility [10–12]. Diagnosis of HCM was historically made based on gross necropsy examination documenting severely thickened left ventricular walls with or without prominent papillary muscles [12]. However, HCM could also be diagnosed by antemortem echocardiographic examination assisted by complimentary electrocardiographic, radiographic and cardiac biomarker analyses [10, 42]. Therefore, precise reference intervals are essential to accurately diagnose HCM using echocardiographic examination. In the present study, the reference intervals of left ventricular wall thickness obtained by the conventional method were different with clinical significance to the ones reported in the previous studies [1, 2, 10]. In addition, allometric scaling analysis revealed that the reference interval is highly dependent on the weight and age, while less dependent on sex. Diagnosis of HCM and other cardiac diseases in rhesus macaques therefore should be done based on the allometric scaling prediction intervals reported in this study. This technique will provide the best opportunity to accurately diagnose this condition and perhaps aid in understanding this non-human primate model of HCM. Future studies should aim to utilize these reference intervals to determine their sensitivity and specificity for diagnosis of various cardiac diseases in rhesus macaques when compared to other methods such as post-mortem pathologic diagnosis.

There are a few limitations to report in this study. One limitation is a lack of complete physical examination and biochemical analysis concurrently performed with echocardiographic examination. Although semiannual to annual examination was performed on these animals and they were deemed to be healthy without any health concerns at the time before sedatives are administered, the possibility of having subclinical systemic disease could not be completely ruled out. This is however unlikely to have a serious impact on the reference intervals and is minimized by the enrollment of a large sample size. In addition, systolic BP measurement was not performed in all rhesus macaques enrolled in the study due to later implementation of routine BP protocol during echocardiographic examination. This is also less likely to impact the findings of this study with a lack of other clinical and echocardiographic signs consistent with systemic hypertension. The impact of possible systemic hypertension is also minimized by a large sample size. Finally, the established reference intervals of various echocardiographic parameters have not been used in different research facilities. Since animals are often inbred in a facility, echocardiographic findings could be different in animals in other facilities. However, due to the fact that rhesus macaques are often exchanged among facilities to avoid development of serious health problems due to inbreeding, it is unlikely to find significant differences in the reference intervals of echocardiographic parameters in clinically healthy rhesus macaques from different research facilities. Nevertheless, further study should be performed with multi-center settings to prove that the reference intervals established in this study can be applied accurately at other facilities and used to diagnose cardiac diseases such as HCM. Lastly, in all rhesus macaques, echocardiographic examination was performed under sedation with ketamine, and the effect of sedatives on echocardiographic parameters should not be ignored. Thus, reference intervals of these parameters under different conditions such as in awake animals or those sedated with a different protocol could differ from those reported in the present study.

## Conclusions

This study is the first to report allometrically scaled echocardiographic reference intervals in a large population. It is also the first to accurately describe the reference intervals with regard to the impact of age and sex in the population. These reference intervals will assist clinicians and researchers to accurately determine the cardiac status of rhesus macaques under ketamine sedation. In addition, mild valve regurgitation is not uncommon in clinically healthy rhesus macaques presumably due to sedative effect or mild valve degeneration. Ultimately, the proposed reference intervals make it possible to accurately identify cardiovascularly healthy rhesus macaques for use as control animals in translational research.

## Methods

### Subjects and housing

This study was conducted at the CNPRC which is the United States Department of Agriculture-registered and the Association for Assessment and Accreditation of Laboratory Animal Care-accredited facility. The CNPRC maintains an approval from the Institutional Animal Care and Use Committee of the University of California-Davis and Public Health Services Assurance. All rhesus macaques enrolled in the study were housed in the CNPRC. Echocardiographic examination and blood pressure measurement under sedation were implemented as a part of routine examinations in the CNPRC, and these examinations were performed to all healthy rhesus macaques before assigned to other experiments, allocations, and transportation to other facilities. All rhesus macaques were returned to their cages after the examination once they were fully recovered from sedation. None of animals were euthanized for completion of this study.

All rhesus macaques at this facility are taken care in accordance with the Animal Welfare Act and Guide for the Care and Use of Laboratory Animals [43]. Outdoor rhesus macaques are all housed as groups in rectangular enclosures sized 0.5-acre. Most of rhesus macaques housed indoor are paired in stainless steel cages sized based on the regulation for primary cage-space limitation. Some of indoor rhesus macaques are housed individually in the same indoor condition. All rhesus macaques are managed with environmental enrichment. They were fed primate chow (LabDiet Monkey Diet 5047, Purina Mills International, St Louis, MO) with vegetables and fruits supplement. Water is provided using automatic watering devise to animals without any restriction to access. Room lighting in the indoor room for the indoor rhesus macaques is automatically controlled with alternating 12 h:12 h light and darkness. Complete physical examination and blood tests including complete blood count analysis and serum biochemistry are performed accordingly. Tuberculosis testing and dental prophylaxis are also performed accordingly. When health issues are noted, these rhesus macaques were transferred to a separated colony and excluded from the present study. All rhesus macaques were also monitored for possible viral infections (herpes B virus, simian type D retrovirus, simian immunodeficiency virus, and simian T-lymphotropic virus).

### Sedation

Ketamine hydrochloride (10 mg/kg IM; Ketaject, Phoenix Pharmaceutical, St. Joseph, MO) was given within five to 10 min prior to echocardiographic examination and blood pressure measurement for sedation for all animals. If necessary, an additional dose of ketamine (5–10 mg/kg IM) was given during echocardiographic assessment to maintain appropriate sedation.

### Echocardiographic measurement

Echocardiographic examination was performed using one of two echocardiographic ultrasound devices (Affiniti 50, Phillips, Best, Netherland, and CX50 Ultrasound System, Phillips, Best, Netherlands) with a 4- to 12-mHz sector-array transducer or a 1- to 5-mHz sector-array transducer. Animals were positioned in right and left lateral recumbency subsequently during the procedure, and 2D and M-mode echocardiography with color and spectral Doppler was performed. In this study, the leading-edge to leading-edge method was employed, and three consecutive measurements were saved for each echocardiographic parameter. All results were analyzed by an author (YU) and reviewed by an ACVIM board-certified cardiologist (JS) using an offline software (Syngo Dynamics, Siemens, Erlangen, Germany) in accordance with the guidelines [44]. After comleting measurement and assessment, rhesus macaques without any significant cardiac abnormalities and changes were selected as control animals from the echocardiographic database developed by the authors (YU, JS).

**Fig. 1:**
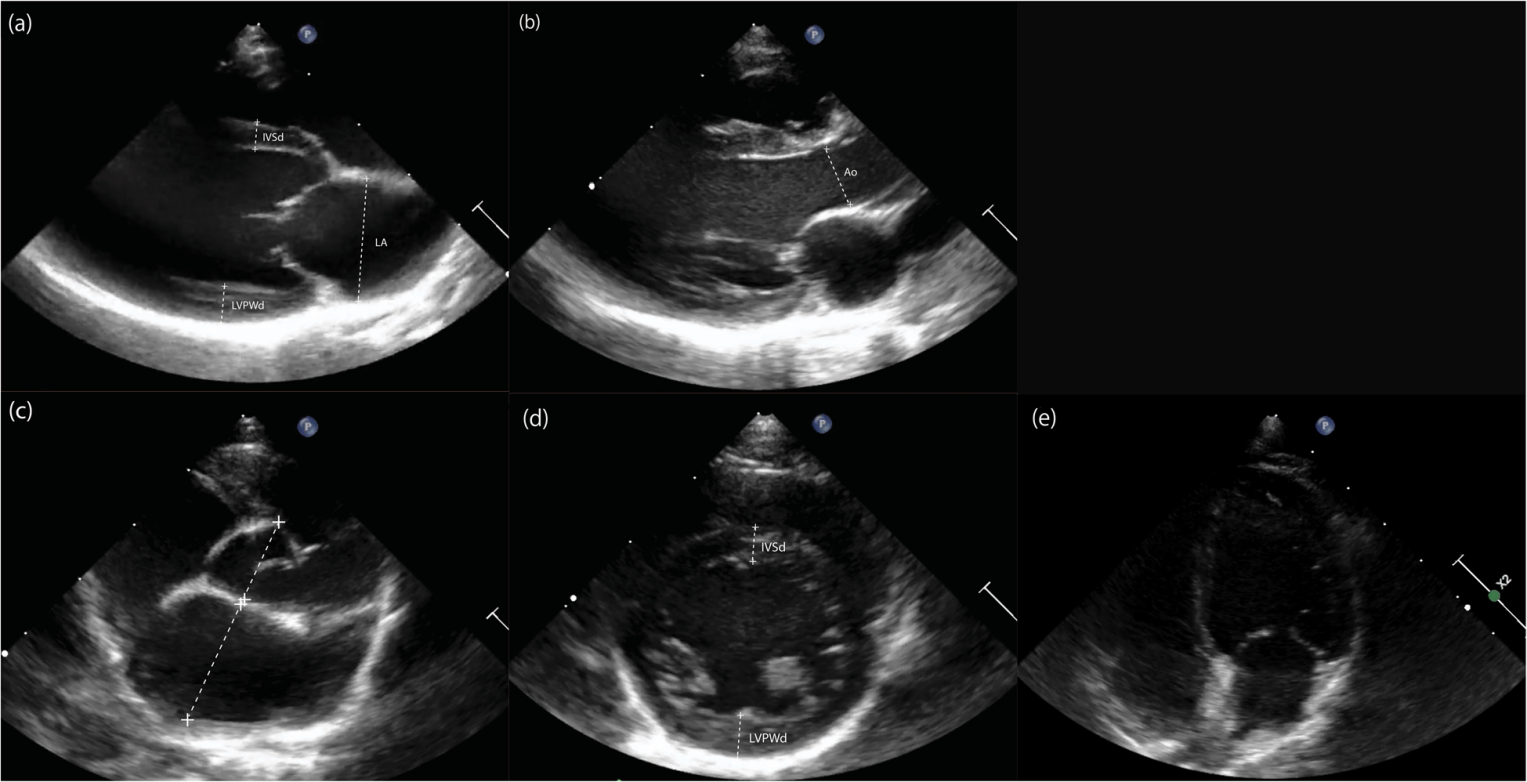
Examples of two-dimensional echocardiographic images. **a** Right parasternal long-axis four-chamber view, **b** right parasternal long-axis left ventricular outflow view, **c** right parasternal short-axis view of the heart vase with aorta, **d** right parasternal short-axis left ventricle with papillary muscles view, **e** left parasternal apical four-chamber view

**Fig. 2:**
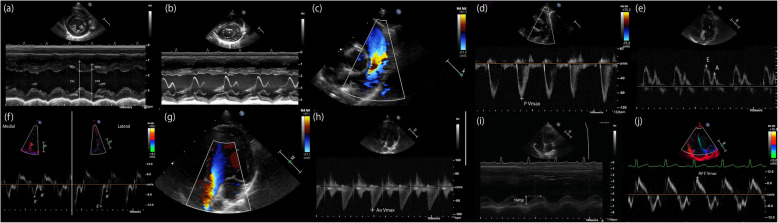
Examples of M-mode and Doppler echocardiographic image. **a** M-mode image of right parasternal short-axis left ventricle with papillary muscle view, **b** M-mode image of right parasternal short-axis mitral valve view, **c** color Doppler image with pulmonary flow in right parasternal short-axis view from heart base with pulmonary artery, **d** pulsed-wave spectral Doppler image with pulmonary flow in right parasternal short-axis view from heart base with pulmonary artery, **e** pulsed-wave spectral Doppler image of mitral flow from left parasternal apical four-chamber view, **f** color-tissue Doppler image with medial and lateral mitral annulus motions from left apical 4-chamber view, **g** color Doppler image of aortic outflow from left apical five-chamber view, **h** continuous-wave spectral Doppler image of aortic outflow from left parasternal five-chamber view **i** M-mode image of tricuspid annular plane systolic excursion from left apical 4-chamber view optimized for the right cardiac chambers, **j** color-tissue Doppler image with longitudinal myocardial motion at the lateral tricuspid annulus from left apical four-chamber view optimized for the right cardiac chambers

Complete echocardiographic examination was performed as previously reported as a part of routine screening and for other ongoing projects by a veterinary cardiologist (JS) or a cardiology research fellow (YU) under the direction of a veterinary cardiologist [3]. Briefly, on right parasternal long-axis four-chamber views, left atrial diameter in diastole (LA [la]) and aortic root diameter in diastole (Ao [la]) were acquired (Fig. 1a and b). On right parasternal short-axis views, LA (LA [sa]) and Ao (Ao [sa]) were acquired at the level of the aortic valve (Fig. 1c). The interventricular septal thickness during diastole (IVSd) and left ventricular posterior wall thickness in diastole (LVPWd) were obtained from the two-dimensional (2D) right parasternal long-axis and short-axis 2-chamber views, and the maximal thickness of these parameters were reported as IVSd (2D) and LVPWd in this study (2D) (Fig. 1a and d). The interventricular septal thickness during systole (IVSs) and diastole (IVSd), left ventricular posterior wall thickness in systole (LVPWs) and diastole (LVPWd), left ventricular internal diameter during systole (LVDs) and diastole (LVDd), and mitral valve E-point to septum separation (EPSS) were measured from the right parasternal short-axis M-mode views at the chordae level (Figs. 1d, 2a, and b). From the right parasternal short-axis right ventricular outflow tract view at the level of the aortic valve, peak pulmonary flow velocity (PV Vmax) and its acceleration time (PV AT), and ejection time (PV ET) were obtained. The sample gate using the pulsed-wave spectral Doppler technique was positioned immediately distal to the pulmonic valve (Fig. 2c and d). On the left parasternal apical four-chamber view, passive early filling (E wave) and atrial contraction later filling (A wave) velocities were obtained (Fig. 1e). The sample gate using the continuous spectral Doppler technique was positioned at the tips of the mitral valve leaflets when they were completely open (Fig. 2e). Color-tissue Doppler imaging (TDI) was performed to obtain free-wall (lateral) and septal (medial) mitral annulus motions from the left apical 4-chamber view. Peak velocities were measured in early (E’ [medial] and E’ [lateral]) and late diastole (A’ [lateral] and A’ [lateral]) (Fig. 2f). Aortic flow profile with maximal aortic flow velocity (Ao Vmax) was obtained from left parasternal apical aortic outflow view with parallel alignment to the aorta using the continuous spectral Doppler technique (Fig. 2g and h) [45, 46]. Acceptable parallel alignment of the Doppler gate was possible in all rhesus macaques, no angle corrections were performed. Using the same images, isovolumic relaxation time (LV IVRT) and mitral E deceleration time (MV DT) were also measured. On the left apical four-chamber view optimized for the right cardiac chambers, tricuspid annular plane systolic excursion (TAPSE) was obtained based on the M-mode by qualifying the maximal longitudinal displacement of the lateral tricuspid valve annulus toward the right ventricular apex during systole. During the measurements, the cursor was placed as parallel as possible to the majority of the right ventricular free wall (Fig. 2i). Pulsed-wave TDI velocities of longitudinal myocardial motion at the lateral tricuspid annulus were also obtained to measure peak systolic annular velocity (RV S′ Vmax) (Fig. 2j).

Using the color Doppler technique, the presence and severity of valve regurgitations were determined on all four cardiac valves as previously performed [3]. Briefly, the severity of aortic regurgitation was categorized as mild (ratio of jet height to left ventricular outflow tract width less than 24%), moderate (25 to 64%), or severe (greater than 65%). The severity of mitral valve regurgitation was categorized as mild (jets occupying less than 29 of left atrial area), moderate (30 to 69%), or severe (more than 70%). The severity of tricuspid valve regurgitation was categorized as mild (right ventricular, right atrium, and vena cava all normal size), moderate (normal or dilated right ventricle, right atrium, or vena cava), or severe (all dilated). Pulmonic regurgitation was quantified as mild (thin narrow origin jet with normal right ventricular size), moderate (wide origin jet with normal or mildly dilated right ventricular size), or severe (wide origin jet with severely dilated right ventricle). Left ventricular outflow tract obstruction (LVOTO) was diagnosed by color Doppler evaluation from right parasternal long axis or left parasternal left ventricular outflow view. Rhesus macaques with moderate or severe valve regurgitation and those with LVOTO were excluded from the present study. Rhesus macaques with no or mild valve regurgitation were enrolled in the present study as long as no other structural and/or functional abnormalities were noted on the echocardiographic examination.

Left ventricular fractional shortening (LV FS) and ejection fraction (LV EF) were calculated as previously described and the animals with LV FS less than 25% and/or LV EF less than 50% were excluded from this study [3].

Diastolic dysfunction was diagnosed by pulsed-wave spectral Doppler trans-mitral filling patterns with a ratio of early to late filling velocities ≤0.9, or with the spectral tissue Doppler lateral or medial peak mitral annular velocity during early and late filling phase ≤0.9 [3, 10, 42]. Rhesus macaques < 18 years old were excluded from this study if they were diagnosed with diastolic dysfunction. Rhesus macaques ≥18 years old with diastolic dysfunction were not excluded from this study as long as there was no concurrent structural or function abnormalities noted on echocardiographic examination [3]. However, the reference intervals of several echocardiographic parameters, including A wave, E wave, E/A, LV IVRT, E:LV IVRT, E’ wave medial and lateral, A’ wave medial and lateral, E’/A’ medial and lateral, E/E’ medial and lateral, were obtained by excluding the cohort of geriatric rhesus macaques that had isolated diastolic dysfunction.

Intra-observer measurement variability was determined by having one of the authors (YU) measure blinded echocardiographic parameters twice on different days from saved echocardiographic images from ten randomly selected rhesus macaques with good image quality. Inter-observer measurement variability was calculated by having two of the authors (YU, LD) measure all echocardiographic parameters, while they are blinded to each other’s measurements.

### Blood pressure measurement

Under sedation, indirect BP measurement was performed in 181 rhesus macaques using an oscillometric systemic BP measurement device (Cardell 9401, Midmark Corp, Versailles, OH, USA) at the same time of echocardiographic examination. Briefly, animals had a systolic, mean and direct BP measured on the left forelimb while the animal was in right lateral recumbency [47]. BP was measured two to three times ensuring that the displayed heart rate on the BP measurement device was confirmed to match the heart rate obtained on echocardiogram and all obtained values were averaged.

### Statistical analysis

This study is a prospective observational study to establish references intervals for echocardiographic parameters on healthy rhesus macaques. 823 rhesus macaques were enrolled in the study when echocardiographic examination was performed as a part of routine examination before assigning to experiments, allocation, and transportation. All data obtained were acquired by the authors (YU, JS) between January 2015 and November 2019 at the CNPRC.

The mean percent coefficient of variation (CV) was calculated for intraobserver and interobserver measurement variabilities using an equation: CV = (SD of the measurement/average of measurement) × 100.

D’Agostino-Pearson test was performed for testing normality of continuous data. Descriptive statistics (mean, SD, and range) was provided as mean with SD or median with interquartile rage for normally distributed parameters and for non-normally distributed parameters, respectively. Outliers were determined and removed by performing post-hoc Tukey test, and double-sided 95% reference intervals were established. Ninety percent confidence interval for each reference limit was determined without the robust method [16].

Pairwise Pearson correlation analysis with animal characteristics (BW, age, sex, HR, and systolic and mean BP) and echocardiographic variables were conducted. The degree of correlation was determined with *r* as weak correlation with *r* < 0.3, moderate correlation with 0.3 ≤ *r* ≤ 0.5, strong correlation with *r* > 0.5.

Multiple regression analysis was conducted between all animal characteristics and the echocardiographic parameters. A model was performed stepwise using BW (kg), age (in days), heart rate (HR; bpm), and sex. The coefficient of the linear association with each echocardiographic parameter and its associated *p*-value was obtained. The coefficients represent the mean change in parameters for an increase of one unit of the explanatory variable while other variables are kept constant.

Linear echocardiographic variables were normalized to BW (kg) based on the constants from power equation, Y = aX^b^ or allometric scaling. In this equation, a, b, Y, and X represent proportionality constant, scaling exponent, and linear echocardiographic parameter, and BW, respectively. Linear regression analysis was then conducted on log10(BW) versus log10 (echo parameter) for each echocardiographic parameter. This process produced the equation, log(Y) = log (a) + b x log(x), where b and a represent the slope and antilog Y-intercept, respectively. The constant (a_c_) based on the formula: a_c_ = log_10_^− 1^[log(a) +/− t x S_x,y_] determined prediction intervals, where a, t, and S_x,y_ represent the proportionality constant, desired Student’s t-statistic for n-2 degrees of freedom, and the standard error of the Y estimate, respectively.

Statistical analysis was performed using commercially available softwares (MedCalc version 12.7.4, MedCalc Software, Ostend, Belgium and Stata Corporation v15.1, College Station, TX). A *P*-value of < 0.05 was considered as significant for all analyses.

## Supplementary information

**Additional file 1: Supplement Table 1.** Echocardiographic parameters showing significant correlations with age, body weight, heart rate, or sex listed as correlation coefficient (r) with the P-value in parenthesis. **Supplement Table 2.** Results of multiple linear regression analysis of echocardiographic parameters with age, body weight, heart rate, or sex. **Supplement Table 3.** Body weight-based means and 95% prediction intervals of 2D and M-mode echocardiographic parameters derived from allometric scaling parameters in 421 male rhesus macaques. **Supplement Table 4.** Body weight-based means and 95% prediction intervals of 2D and M-mode echocardiographic parameters derived from allometric scaling parameters in 402 female rhesus macaques. **Supplement Table 5.** Body weight-based means and 95% prediction intervals of 2D and M-mode echocardiographic parameters derived from allometric scaling parameters in 328 rhesus macaques in the range of 5 months to 4 years old. **Supplement Table 6.** Body weight-based means and 95% prediction intervals of 2D and M-mode echocardiographic parameters derived from allometric scaling parameters in 264 rhesus macaques in the range of 5 years to 9 years old. **Supplement Table 7.** Body weight-based means and 95% prediction intervals of 2D and M-mode echocardiographic parameters derived from allometric scaling parameters in 137 rhesus macaques in the range of 10 months to 14 years old. **Supplement Table 8.** Body weight-based means and 95% prediction intervals of 2D and M-mode echocardiographic parameters derived from allometric scaling parameters in 94 rhesus macaques over 15 years old.

## Data Availability

The datasets used and/or analyzed during the current study are available from the corresponding author on reasonable request.

## Abbreviations

A wave: Atrial contraction late filling velocity
A’ wave: Late diastolic mitral annulus motion
Ao (la): Aortic root diameter from long-axis view
Ao (sa): Aortic root diameter from short-axis view
Ao Vmax: Peak velocity for aortic flow
BP: Blood pressure
BW: Body weight
CNPRC: California National Primate Research Center
CV: Coefficient of variation
DF: Degree of freedom
E wave: Passive filling early filling velocity
E’ wave: Early diastolic mitral annulus motion
EPSS: E-point septal separation
HCM: Hypertrophic cardiomyopathy
HR: Heart rate
IVSd: Interventricular septal wall thickness during diastole
IVSs: Interventricular septal wall thickness during systole
LA (la): Left atrial diameter in diastole from long-axis view
LA (sa): Left atrial diameter in diastole from short-axis view
LVDd: Left ventricular internal diameter in diastole
LVDs: Left ventricular internal diameter in systole
LV EF: Left ventricular ejection fraction
LV FS: Left ventricular fractional shortening
LV IVRT: Left ventricular isovolumic relaxation time
LVOTO: Left ventricular outflow tract obstruction
LVPWd: Left ventricular posterior wall thickness during diastole
LVPWs: Left ventricular posterior wall thickness during systole
MV DT: Left ventricular deceleration time
PV AT: Pulmonary valve acceleration time
PV ET: Pulmonary valve ejection time
PV Vmax: Peak velocity for pulmonary flow
RV S′ Vmax: Tricuspid peak systolic annular velocity
SAM: Systolic anterior motion
SD: Standard deviation
TAPSE: Tricuspid annular plane systolic excursion
TDI: Color tissue Doppler image
2D: Two-dimensional
