# Supplementary material for: Echocardiographic reference intervals with allometric scaling of 823 clinically healthy rhesus macaques (Macaca mulatta)
Source: BMC Vet Res. 2020 Sep 22;16:348. doi: 10.1186/s12917-020-02578-y (PMC7510309; doi:10.1186/s12917-020-02578-y)
Supplement: Supplementary file 1 — Additional file 1: Supplement Table 1. Echocardiographic parameters showing significant correlations with age, body weight, heart rate, or sex listed as correlation coefficient (r) with the P-value in parenthesis. Supplement Table 2. Results of multiple linear regression analysis of echocardiographic parameters with age, body weight, heart rate, or sex. Supplement Table 3. Body weight-based means and 95% prediction intervals of 2D and M-mode echocardiographic parameters derived from allometric scaling parameters in 421 male rhesus macaques. Supplement Table 4. Body weight-based means and 95% prediction intervals of 2D and M-mode echocardiographic parameters derived from allometric scaling parameters in 402 female rhesus macaques. Supplement Table 5. Body weight-based means and 95% prediction intervals of 2D and M-mode echocardiographic parameters derived from allometric scaling parameters in 328 rhesus macaques in the range of 5 months to 4 years old. Supplement Table 6. Body weight-based means and 95% prediction intervals of 2D and M-mode echocardiographic parameters derived from allometric scaling parameters in 264 rhesus macaques in the range of 5 years to 9 years old. Supplement Table 7. Body weight-based means and 95% prediction intervals of 2D and M-mode echocardiographic parameters derived from allometric scaling parameters in 137 rhesus macaques in the range of 10 months to 14 years old. Supplement Table 8. Body weight-based means and 95% prediction intervals of 2D and M-mode echocardiographic parameters derived from allometric scaling parameters in 94 rhesus macaques over 15 years old. [file 12917_2020_2578_MOESM1_ESM.docx]

**Supplement Table 1.** Echocardiographic parameters showing significant correlations with age, body weight, heart rate, or sex listed as correlation coefficient (*r)* with the *P*-value in parenthesis.

| Echo parameters |  | WT (kg) | Age (days) | Sex | HR (bpm) | Systolic BP | Mean BP |
| --- | --- | --- | --- | --- | --- | --- | --- |
| LA (sa) | r | 0.68 | 0.35 | -0.2 | -0.35 | 0.21 |  |
|  | *p*-value | <0.0001 | <0.0001 | <0.0001 | <0.0001 | 0.0057 |  |
| Ao (sa) | r | 0.7 | 0.54 | -0.084 | -0.26 | 0.3 | 0.27 |
|  | *p*-value | <0.0001 | <0.0001 | 0.018 | <0.0001 | <0.0001 | 0.0002 |
| LA/Ao (sa) | r | -0.073 | -0.24 | -0.11 | -0.11 |  |  |
|  | *p*-value | 0.037 | <0.0001 | 0.0021 | 0.017 |  |  |
| LA (la) | r | 0.64 | 0.34 | -0.21 | -0.32 | 0.16 |  |
|  | *p*-value | <0.0001 | <0.0001 | <0.0001 | <0.00001 | 0.036 |  |
| Ao (la) | r | 0.71 | 0.5 | -0.12 | -0.27 | 0.16 |  |
|  | *p*-value | <0.0001 | <0.0001 | 0.0004 | <0.0001 | 0.033 |  |
| LA/Ao | r | -0.18 | -0.22 |  |  |  |  |
|  | *p*-value | <0.0001 | <0.0001 |  |  |  |  |
| IVSd (2D) | r | 0.53 | 0.39 | -0.18 |  | 0.16 | 0.17 |
|  | *p*-value | <0.0001 | <0.0001 | <0.0001 |  | 0.027 | 0.02 |
| LVPWd (2D) | r | 0.63 | 0.44 | -0.18 | -0.16 | 0.19 |  |
|  | *p*-value | <0.0001 | <0.0001 | <0.0001 | 0.005 | 0.012 |  |
| IVSd (M-mode) | r | 0.5 | 0.29 | -0.22 | -0.2 |  |  |
|  | *p*-value | <0.0001 | <0.0001 | <0.0001 | <0.0001 |  |  |
| LVDd (M-mode) | r | 0.67 | 0.26 | -0.22 | -0.45 |  |  |
|  | *p*-value | <0.0001 | <0.0001 | <0.0001 | <0.0001 |  |  |
| LVPWd (M-mode) | r | 0.62 | 0.43 | -0.15 | -0.15 | 0.18 | 0.19 |
|  | *p*-value | 0.0001 | <0.0001 | <0.0001 | 0.0011 | 0.017 | 0.0099 |
| IVSs (M-mode) | r | 0.53 | 0.35 | -0.13 | -0.12 | 0.16 |  |
|  | *p*-value | <0.0001 | <0.0001 | 0.0003 | 0.0064 | 0.032 |  |
| LVDs (M-mode) | r | 0.46 | 0.088 | -0.28 | -0.42 |  |  |
|  | *p*-value | <0.0001 | 0.012 | <0.0001 | <0.0001 |  |  |
| LVPWs (M-mode) | r | 0.64 | 0.46 | -0.093 | -0.13 | 0.25 | 0.21 |
|  | *p*-value | <0.0001 | <0.0001 | 0.0077 | 0.0033 | 0.0009 | 0.0054 |
| LV FS | r | 0.43 | 0.19 | 0.2 | 0.12 | 0.2 | 0.18 |
|  | *p*-value | <0.0001 | <0.0001 | <0.0001 | 0.0088 | 0.0063 | 0.013 |
| LV EF | r | 0.55 | 0.16 | 0.22 | 0.15 | 0.19 | 0.19 |
|  | *p*-value | <0.0001 | <0.0001 | <0.0001 | 0.005 | 0.011 | 0.013 |
| EPSS | r | 0.36 | 0.11 | -0.21 | -0.29 |  |  |
|  | *p*-value | <0.0001 | 0.0041 | <0.0001 | <0.0001 |  |  |
| LV IVRT | r | 0.13 | 0.1 | -0.07 | -0.46 |  |  |
|  | *p*-value | 0.0002 | 0.0033 | 0.047 | <0.0001 |  |  |
| E: LV IVRT | r | -0.32 | -0.36 |  | 0.28 |  |  |
|  | *p*-value | <0.0001 | <0.0001 |  | <0.0001 |  |  |
| E' wave medial | r | -0.17 | -0.26 |  | 0.22 | 0.17 | 0.22 |
|  | *p*-value | <0.0001 | <0.0001 |  | <0.0001 | 0.029 | 0.0044 |
| A' wave medial | r |  | 0.2 | 0.19 | 0.32 | 0.27 | 0.29 |
|  | *p*-value |  | <0.0001 | <0.0001 | <0.0001 | 0.0008 | 0.0002 |
| E/E' medial | r | -0.21 | -0.21 | -0.19 |  |  |  |
|  | *p*-value | <0.0001 | <0.0001 | <0.0001 |  |  |  |
| E' /A' medial | r | -0.21 | -0.42 | -0.28 | -0.3 | -0.17 |  |
|  | *p*-value | <0.0001 | <0.0001 | <0.0001 | <0.0001 | 0.029 |  |
| E' wave lateral | r | -0.16 | -0.22 |  | 0.11 |  |  |
|  | *p*-value | <0.0001 | <0.0001 |  | 0.018 |  |  |
| A' wave lateral | r |  | 0.36 | 0.3 | 0.18 | 0.28 | 0.27 |
|  | *p*-value |  | <0.0001 | <0.0001 | 0.0001 | 0.0004 | 0.0004 |
| E/E' lateral | r | -0.17 | -0.13 | -0.1 |  |  |  |
|  | *p*-value | <0.0001 | 0.0009 | 0.013 |  |  |  |
| E'/A' lateral | r | -0.19 | -0.51 | -0.31 | -0.19 | -0.18 | -0.18 |
|  | *p*-value | <0.0001 | <0.0001 | <0.0001 | 0.0001 | 0.023 | 0.021 |
| Ao Vmax | r | -0.17 | -0.3 |  | 0.18 | 0.18 | 0.16 |
|  | *p*-value | <0.0001 | <0.0001 |  | 0.0001 | 0.016 | 0.037 |
| MV DT | r | 0.21 | 0.077 |  | -0.51 |  |  |
|  | *p*-value | <0.0001 | 0.046 |  | <0.0001 |  |  |
| E wave | r | -0.42 | -0.51 | -0.154 | 0.094 |  |  |
|  | *p*-value | <0.0001 | <0.0001 | <0.0001 | 0.036 |  |  |
| A wave | r |  | 0.12 | 0.14 | 0.47 | 0.24 | 0.25 |
|  | *p*-value |  | 0.0011 | 0.0001 | <0.0001 | 0.0017 | 0.0011 |
| E/A | r | -0.27 | -0.49 | -0.24 | -0.4 | -0.19 | -0.22 |
|  | *p*-value | <0.0001 | <0.0001 | <0.0001 | <0.0001 | 0.013 | 0.0042 |
| PV Vmax | r | 0.12 |  | -0.08 | 0.2 | 0.18 | 0.19 |
|  | *p*-value | 0.005 |  | <0.0001 | <0.0001 | 0.017 | 0.0089 |
| PV AT | r | 0.1 |  | -0.14 | -0.33 |  | -0.15 |
|  | *p*-value | 0.0041 |  | 0.0001 | <0.0001 |  | 0.041 |
| PV ET | r |  | -0.17 | -0.13 | -0.7 |  |  |
|  | *p*-value |  | <0.0001 | 0.0001 | <0.0001 |  |  |
| PV AT/ET | r | 0.12 | 0.12 |  |  |  |  |
|  | *p*-value | 0.0005 | 0.0008 |  |  |  |  |
| TAPSE | r | 0.17 |  |  | -0.27 |  |  |
|  | *p*-value | <0.0001 |  |  | <0.0001 |  |  |
| RV S' Vmax | r | 0.18 | 0.23 |  | 0.15 | 0.22 | 0.21 |
|  | *p*-value | 0.0002 | <0.0001 |  | 0.004 | 0.0058 | 0.0064 |

A wave, passive filling velocity; A’ wave, late diastolic mitral annulus motion; Ao (la), aortic root diameter from long-axis view; Ao (sa), aortic root diameter from short-axis view; E’, early diastolic mitral annulus motion; EPSS, E-point septal separation; LA (la), left atrial diameter from long-axis view; LA (sa), left atrial diameter from short-axis view; LVd, left ventricular internal diameter in diastole; LV IVRT, left ventricular isovolumic relaxation time; LVs, left ventricular internal diameter in systole; LVPWd, left ventricular posterior wall thickness in diastole; MV DT, left ventricular deceleration time; PV AT, pulmonary valve acceleration time; PV ET, pulmonary valve ejection time; Ao Vmax, peak velocity for aortic flow; PV Vmax, peak velocity for pulmonary flow; TAPSE, tricuspid annular plane systolic excursion; RV S’ Vmax, tricuspid peak systolic annular velocity

*Only the parameters with significant correlations are listed in this table.

**Supplement Table 2.** Results of multiple linear regression analysis of echocardiographic parameters with age, body weight, heart rate, or sex.

|  |  | HR | WT | Age | Sex | F-value | DF | P-value | R^2^ |
| --- | --- | --- | --- | --- | --- | --- | --- | --- | --- |
| LA (sa) | Coefficients | -0.0025 | 0.051 |  |  | 130.5 | 493 | <0.0001 | 0.5144 |
|  | P-value | <0.0001 | <0.0001 |  |  |  |  |  |  |
| Ao (sa) | Coefficients | -0.0012 | 0.032 | 0.000035 |  | 153.7 | 493 | <0.0001 | 0.555 |
|  | P-value | <0.0001 | <0.0001 | <0.00001 |  |  |  |  |  |
| LA/Ao (sa) | Coefficients | -0.00071 |  | -0.000026 |  | 8.05 | 493 | <0.0001 | 0.0613 |
|  | P-value | 0.035 |  | <0.0001 |  |  |  |  |  |
| LA (la) | Coefficients | -0.0025 | 0.05 | 0.000017 |  | 98.52 | 495 | <0.0001 | 0.4433 |
|  | P-value | <0.0001 | <0.0001 | 0.045 |  |  |  |  |  |
| Ao (la) | Coefficients | -0.00088 | 0.028 | 0.00002 | -0.026 | 167.2 | 495 | <0.0001 | 0.5746 |
|  | P-value | <0.0001 | <0.0001 | <0.0001 | 0.016 |  |  |  |  |
| LA/Ao | Coefficients |  |  | -0.000022 |  | 6.17 | 495 | 0.0001 | 0.0475 |
|  | P-value |  |  | 0.027 |  |  |  |  |  |
| IVSd (2D) | Coefficients |  | 0.11 | 0.00018 | -0.41 | 57.95 | 499 | <0.0001 | 0.3189 |
|  | P-value |  | <0.0001 | <0.0001 | <0.0001 |  |  |  |  |
| LVPWd (2D) | Coefficients |  | 0.14 | 0.00014 | -0.31 | 100.7 | 495 | <0.0001 | 0.4486 |
|  | P-value |  | <0.0001 | <0.0001 | <0.0001 |  |  |  |  |
| IVSd (M-mode) | Coefficients | -0.00037 | 0.0099 | 0.0000098 | -0.044 | 49.39 | 496 | <0.0001 | 0.2849 |
|  | P-value | 0.015 | <0.0001 | 0.001 | <0.0001 |  |  |  |  |
| LVDd (M-mode) | Coefficients | -0.0047 | 0.07 |  |  | 175.2 | 496 | <0.0001 | 0.5856 |
|  | P-value | <0.0001 | <0.0001 |  |  |  |  |  |  |
| LVPWd (M-mode) | Coefficients |  | 0.016 | 0.000013 | -0.025 | 91.05 | 496 | <0.0001 | 0.4234 |
|  | P-value |  | <0.0001 | <0.0001 | 0.003 |  |  |  |  |
| IVSs (M-mode) | Coefficients |  | 0.017 | 0.000013 | -0.035 | 41.83 | 496 | <0.0001 | 0.2522 |
|  | P-value |  | <0.0001 | 0.006 | 0.009 |  |  |  |  |
| LVDs (M-mode) | Coefficients | -0.0036 | 0.042 |  | -0.098 | 85.39 | 496 | <0.0001 | 0.4078 |
|  | P-value | <0.0001 | <0.0001 |  | <0.0001 |  |  |  |  |
| LVPWs (M-mode) | Coefficients |  | 0.026 | 0.000018 |  | 104.3 | 496 | <0.0001 | 0.4568 |
|  | P-value |  | <0.0001 | <0.0001 |  |  |  |  |  |
| LV FS | Coefficients | 0.033 | 0.16 |  | 2.61 | 11 | 496 | <0.0001 | 0.0815 |
|  | P-value | 0.012 | 0.22 |  | <0.0001 |  |  |  |  |
| LV EF | Coefficients | 0.046 |  |  | 3.53 | 13.06 | 500 | <0.0001 | 0.0953 |
|  | P-value | 0.003 |  |  | <0.0001 |  |  |  |  |
| EPSS | Coefficients | -0.00085 | 0.011 |  | -0.026 | 32.13 | 481 | <0.0001 | 0.2109 |
|  | P-value | <0.0001 | <0.0001 |  | 0.009 |  |  |  |  |
| LV IVRT | Coefficients | -0.18 |  | 0.0012 | -2.055 | 40.52 | 494 | <0.0001 | 0.247 |
|  | P-value | <0.0001 |  | <0.0001 | 0.022 |  |  |  |  |
| E: LV IVRT | Coefficients | 0.000064 | -0.00021 | 0.000000097 |  | 32.42 | 492 | <0.0001 | 0.2086 |
|  | P-value | <0.0001 | 0.039 | <0.0001 |  |  |  |  |  |
| E' wave medial | Coefficients | 0.00016 |  | 0.0000028 |  | 12.39 | 478 | <0.0001 | 0.0939 |
|  | P-value | <0.0001 |  | <0.0001 |  |  |  |  |  |
| A' wave medial | Coefficients | 0.00031 |  | 0.0000032 |  | 18.8 | 466 | <0.0001 | 0.1389 |
|  | P-value | <0.0001 |  | 0.001 |  |  |  |  |  |
| E/E' medial | Coefficients | -0.018 | -0.27 |  |  | 15.53 | 475 | <0.0001 | 0.1156 |
|  | P-value | 0.003 | <0.0001 |  |  |  |  |  |  |
| E' /A' medial | Coefficients | -0.0045 |  | -0.000098 | -0.08 | 40.66 | 467 | <0.0001 | 0.2583 |
|  | P-value | <0.0001 |  | <0.0001 | 0.032 |  |  |  |  |
| E' wave lateral | Coefficients | 0.00021 |  |  |  | 4.78 | 440 | 0.0009 | 0.0416 |
|  | P-value | 0.036 |  |  |  |  |  |  |  |
| A' wave lateral | Coefficients | 0.00014 |  | 0.0000047 | 0.0057 | 27.14 | 422 | <0.0001 | 0.2061 |
|  | P-value | 0.001 |  | <0.0001 | 0.007 |  |  |  |  |
| E/E' lateral | Coefficients |  | -0.1 |  |  | 5.98 | 438 | 0.0001 | 0.0518 |
|  | P-value |  | 0.028 |  |  |  |  |  |  |
| E'/A' lateral | Coefficients | -0.0039 |  | -0.00015 | -0.13 | 44.09 | 422 | <0.0001 | 0.2967 |
|  | P-value | <0.0001 |  | <0.0001 | 0.009 |  |  |  |  |
| Ao Vmax | Coefficients | 0.0015 |  | -0.00027 |  | 9.93 | 487 | <0.0001 | 0.0754 |
|  | P-value | <0.0001 |  | <0.0001 |  |  |  |  |  |
| MV DT | Coefficients | -0.51 | 1.059 |  |  | 40.47 | 426 | <0.0001 | 0.2754 |
|  | P-value | <0.0001 | 0.009 |  |  |  |  |  |  |
| E wave | Coefficients |  | -0.014 | -0.00032 | -0.046 | 45.54 | 496 | <0.0001 | 0.2702 |
|  | P-value |  | <0.0001 | <0.0001 | 0.006 |  |  |  |  |
| A wave | Coefficients | 0.0029 |  |  |  | 34.79 | 470 | <0.0001 | 0.2284 |
|  | P-value | <0.0001 |  |  |  |  |  |  |  |
| E/A | Coefficients | -0.007 | -0.022 | -0.000077 | -0.12 | 68.4 | 470 | <0.0001 | 0.3679 |
|  | P-value | <0.0001 | 0.001 | <0.0001 | 0.002 |  |  |  |  |
| PV Vmax | Coefficients | 0.0016 | 0.014 | -0.000016 | 0.059 | 11.3 | 492 | <0.0001 | 0.0841 |
|  | P-value | <0.0001 | <0.0001 | 0.007 | 0.001 |  |  |  |  |
| PV AT | Coefficients | -0.23 |  |  | -5.56 | 17.52 | 492 | <0.0001 | 0.1247 |
|  | P-value | <0.0001 |  |  | 0.002 |  |  |  |  |
| PV ET | Coefficients | -0.7 |  |  |  | 123.1 | 496 | <0.00001 | 0.5002 |
|  | P-value | <0.0001 |  |  |  |  |  |  |  |
| PV AT/ET | Coefficients |  |  | 0.0000045 | -0.023 | 4.69 | 492 | 0.001 | 0.0367 |
|  | P-value |  |  | 0.049 | <0.0001 |  |  |  |  |
| TAPSE | Coefficients | -0.0024 | 0.015 |  |  | 12.72 | 477 | <0.0001 | 0.0964 |
|  | P-value | <0.0001 | 0.001 |  |  |  |  |  |  |
| RV S' Vmax | Coefficients | 0.00017 | 0.0011 | 0.0000019 |  | 9.29 | 374 | <0.0001 | 0.0904 |
|  | P-value | 0.001 | 0.026 | 0.047 |  |  |  |  |  |

A wave, passive filling velocity; A’ wave, late diastolic mitral annulus motion; Ao (la), aortic root diameter from long-axis view; Ao (sa), aortic root diameter from short-axis view; E’, early diastolic mitral annulus motion; EPSS, E-point septal separation; LA (la), left atrial diameter from long-axis view; LA (sa), left atrial diameter from short-axis view; LVd, left ventricular internal diameter in diastole; LV IVRT, left ventricular isovolumic relaxation time; LVs, left ventricular internal diameter in systole; LVPWd, left ventricular posterior wall thickness in diastole; PV AT, pulmonary valve acceleration time; PV ET, pulmonary valve ejection time; Ao Vmax, peak velocity for aortic flow; PV Vmax, peak velocity for pulmonary flow; TAPSE, tricuspid annular plane systolic excursion; RV S’ Vmax, tricuspid peak systolic annular velocity; DF, degree of freedom

**Supplement Table 3.** Body weight-based means and 95% prediction intervals of 2D and M-mode echocardiographic parameters derived from allometric scaling parameters in 426 male rhesus macaques.

| Echo parameters | Body weight | | | | | | | | | | | |
| --- | --- | --- | --- | --- | --- | --- | --- | --- | --- | --- | --- | --- |
|  | 1 | 2 | 4 | 6 | 8 | 10 | 12 | 14 | 16 | 18 | 20 | 22 |
| LA (sa) | 1 | 1.2 | 1.5 | 1.7 | 1.8 | 1.9 | 2 | 2.1 | 2.2 | 2.3 | 2.4 | 2.4 |
|  | 0.84-1.22 | 1.02-1.48 | 1.24-1.80 | 1.39-2.02 | 1.51-2.19 | 1.58-2.3 | 1.69-2.45 | 1.77-2.56 | 1.84-2.66 | 1.9-2.75 | 1.96-2.83 | 2.01-2.91 |
| Ao (sa) | 0.7 | 0.9 | 1 | 1.2 | 1.3 | 1.4 | 1.4 | 1.5 | 1.6 | 1.6 | 1.7 | 1.7 |
|  | 0.57-0.59 | 0.71-1.04 | 0.87-1.27 | 0.98-1.42 | 1.06-1.54 | 1.13-1.64 | 1.19-1.73 | 1.24-1.81 | 1.29-1.88 | 1.33-1.94 | 1.37-2 | 1.41-2.05 |
| LA (la) | 1.2 | 1.4 | 1.7 | 1.9 | 2 | 2.1 | 2.2 | 2.3 | 2.4 | 2.5 | 2.6 | 2.6 |
|  | 0.98-1.45 | 1.17-1.73 | 1.4-2.06 | 1.55-2.29 | 1.67-2.46 | 1.76-2.61 | 1.85-2.73 | 1.92-2.84 | 1.99-2.94 | 2.05-3.03 | 2.11-3.12 | 2.16-3.19 |
| Ao (la) | 0.5 | 0.7 | 0.8 | 0.9 | 1 | 1.1 | 1.1 | 1.2 | 1.2 | 1.3 | 1.3 | 1.4 |
|  | 0.44-0.64 | 0.55-0.79 | 0.68-0.98 | 0.76-1.11 | 0.83-1.21 | 0.89-1.21 | 0.94-1.37 | 0.99-1.43 | 1.03-1.49 | 1.07-1.54 | 1.1-1.59 | 1.13-1.64 |
| IVSd (2D) | 0.35 | 0.41 | 0.48 | 0.52 | 0.55 | 0.58 | 0.61 | 0.63 | 0.65 | 0.66 | 0.68 | 0.69 |
|  | 0.27-0.45 | 0.32-0.52 | 0.37-0.61 | 0.41-0.67 | 0.43-0.71 | 0.45-0.75 | 0.47-0.78 | 0.49-0.81 | 0.50-0.83 | 0.52-0.85 | 0.53-0.87 | 0.54-0.89 |
| LVPWd (2D) | 0.36 | 0.43 | 0.51 | 0.57 | 0.61 | 0.64 | 0.67 | 0.7 | 0.72 | 0.75 | 0.77 | 0.79 |
|  | 0.3-0.44 | 0.35-0.52 | 0.42-0.62 | 0.47-0.69 | 0.5-0.74 | 0.53-0.78 | 0.56-0.82 | 0.58-0.85 | 0.6-0.88 | 0.62-0.91 | 0.63-0.93 | 0.65-0.95 |
| IVSd (M-mode) | 0.3 | 0.4 | 0.4 | 0.5 | 0.5 | 0.6 | 0.6 | 0.6 | 0.6 | 0.6 | 0.7 | 0.7 |
|  | 0.24-0.4 | 0.29-0.48 | 0.34-0.57 | 0.38-0.63 | 0.4-0.68 | 0.43-0.71 | 0.45-0.75 | 0.46-0.78 | 0.48-0.8 | 0.49-0.83 | 0.51-0.85 | 0.52-0.87 |
| LVd (M-mode) | 1.2 | 1.5 | 1.8 | 2 | 2.2 | 2.3 | 2.5 | 2.6 | 2.7 | 2.8 | 2.9 | 2.9 |
|  | 1-1.47 | 1.22-1.8 | 1.49-2.19 | 1.67-2.46 | 1.82-2.67 | 1.94-2.84 | 2.04-3 | 2.13-3.13 | 2.22-3.25 | 2.29-3.37 | 2.36-3.47 | 2.43-3.56 |
| LVPWd (M-mode) | 0.3 | 0.4 | 0.5 | 0.5 | 0.6 | 0.6 | 0.6 | 0.7 | 0.7 | 0.7 | 0.7 | 0.7 |
|  | 0.25-0.39 | 0.3-0.48 | 0.37-0.58 | 0.41-0.65 | 0.45-0.71 | 0.48-0.76 | 0.5-0.8 | 0.52-0.83 | 0.54-0.86 | 0.56-0.89 | 0.58-0.92 | 0.59-0.94 |
| IVSs (M-mode) | 0.6 | 0.7 | 0.8 | 0.9 | 1 | 1 | 1.1 | 1.1 | 1.1 | 1.2 | 1.2 | 1.2 |
|  | 0.33-0.56 | 0.4-0.68 | 0.48-0.81 | 0.53-0.91 | 0.58-0.98 | 0.61-1.04 | 0.64-1.09 | 0.67-1.13 | 0.69-1.17 | 0.71-1.21 | 0.73-1.25 | 0.75-1.28 |
| LVs (M-mode) | 0.6 | 0.7 | 0.8 | 0.9 | 1 | 1.1 | 1.1 | 1.2 | 1.2 | 1.2 | 1.3 | 1.3 |
|  | 0.55-1.05 | 0.67-1.28 | 0.81-1.56 | 0.91-1.74 | 0.98-1.89 | 1.05-2.01 | 1.1-2.12 | 1.15-2.21 | 1.2-2.3 | 1.24-2.38 | 1.27-2.45 | 1.31-2.51 |
| LVPWs M-mode) | 0.6 | 0.7 | 0.8 | 0.9 | 1 | 1.1 | 1.2 | 1.2 | 1.3 | 1.3 | 1.4 | 1.4 |
|  | 0.35-0.56 | 0.43-0.7 | 0.53-0.86 | 0.6-0.97 | 0.66-1.06 | 0.7-1.13 | 0.74-1.2 | 0.78-1.25 | 0.81-1.30 | 0.84-1.35 | 0.86-1.4 | 0.89-1.44 |

IVSd, in­terventricular septal thickness in diastole; IVSs, interventricular septal thickness in systole; LVDs, left ventricular internal diameter in systole; LVPWd, left ventricular posterior wall thickness in diastole; LVPWs, left ventricular posterior wall thickness in systole; LA (la), left atrial diameter in diastole from long-axis view; LA (sa), left atrial diameter from short-axis view; TAPSE, tricuspid annular plane systolic excursion

**Supplement Table 4.** Body weight-based means and 95% prediction intervals of 2D and M-mode echocardiographic parameters derived from allometric scaling parameters in 403 female rhesus macaques

| Echo parameters | Body weight | | | | | | | | | | | |
| --- | --- | --- | --- | --- | --- | --- | --- | --- | --- | --- | --- | --- |
|  | 1 | 2 | 4 | 6 | 8 | 10 | 12 | 14 | 16 | 18 | 20 | 22 |
| LA (sa) | 1.1 | 1.3 | 1.5 | 1.7 | 1.8 | 1.9 | 1.9 | 2 | 2.1 | 2.1 | 2.2 | 2.2 |
|  | 0.93-1.37 | 1.08-1.59 | 1.26-1.84 | 1.38-2.01 | 1.46-2.14 | 1.54-2.25 | 1.6-2.34 | 1.65-2.42 | 1.7-2.49 | 1.75-2.56 | 1.79-2.62 | 1.82-2.67 |
| Ao (sa) | 0.9 | 1.1 | 1.2 | 1.3 | 1.3 | 1.4 | 1.5 | 1.5 | 1.6 | 1.7 | 1.7 | 1.8 |
|  | 0.72-1.04 | 0.88-1.27 | 0.99-1.43 | 1.08-1.56 | 1.08-1.56 | 1.16-1.67 | 1.22-1.76 | 1.28-1.84 | 1.33-1.92 | 1.38-1.98 | 1.42-2.05 | 1.46-2.1 |
| LA (la) | 1.2 | 1.4 | 1.7 | 1.8 | 2 | 2.1 | 2.2 | 2.2 | 2.3 | 2.4 | 2.4 | 2.5 |
|  | 0.94-1.53 | 1.11-1.8 | 1.3-2.13 | 1.44-2.35 | 1.54-2.51 | 1.62-2.65 | 1.7-2.77 | 1.76-2.87 | 1.82-2.96 | 1.87-3.05 | 1.92-3.13 | 1.96-3.2 |
| Ao (la) | 0.5 | 0.6 | 0.8 | 0.9 | 1 | 1.1 | 1.1 | 1.2 | 1.3 | 1.3 | 1.4 | 1.4 |
|  | 0.42-0.63 | 0.53-0.79 | 0.66-0.99 | 0.75-1.12 | 0.83-1.23 | 0.89-1.31 | 0.94-1.45 | 0.99-1.47 | 1.03-1.54 | 1.07-1.6 | 1.11-1.65 | 1.14-1.67 |
| IVSd (2D) | 0.3 | 0.36 | 0.44 | 0.49 | 0.54 | 0.57 | 0.6 | 0.63 | 0.65 | 0.68 | 0.7 | 0.72 |
|  | 0.23-0.39 | 0.28-0.47 | 0.34-0.57 | 0.38-0.64 | 0.42-0.69 | 0.44-0.74 | 0.47-0.78 | 0.49-0.81 | 0.51-0.85 | 0.52-0.87 | 0.54-0.9 | 0.55-0.93 |
| LVPWd (2D) | 0.35 | 0.42 | 0.5 | 0.55 | 0.59 | 0.63 | 0.66 | 0.68 | 0.71 | 0.73 | 0.75 | 0.76 |
|  | 0.27-0.5 | 0.33-0.53 | 0.39-0.63 | 0.44-0.7 | 0.47-0.75 | 0.5-0.79 | 0.52-0.83 | 0.54-0.86 | 0.56-0.89 | 0.57-0.92 | 0.59-0.94 | 0.64-0.97 |
| IVSd (M-mode) | 0.3 | 0.4 | 0.4 | 0.5 | 0.5 | 0.5 | 0.5 | 0.6 | 0.6 | 0.6 | 0.6 | 0.6 |
|  | 0.23-0.43 | 0.27-0.5 | 0.34-0.58 | 0.34-0.63 | 0.36-0.67 | 0.38-0.70 | 0.39-0.73 | 0.41-0.75 | 0.42-0.77 | 0.43-0.79 | 0.44-0.81 | 0.45-0.82 |
| LVd (M-mode) | 1.4 | 1.6 | 1.8 | 2 | 2.1 | 2.2 | 2.3 | 2.4 | 2.5 | 2.6 | 2.6 | 2.7 |
|  | 1.13-1.62 | 1.32-1.89 | 1.54-2.2 | 1.68-2.4 | 1.79-2.55 | 1.88-2.68 | 1.95-2.79 | 2.02-2.89 | 2.08-2.97 | 2.13-3.05 | 2.18-3.12 | 2.23-3.19 |
| LVPWd (M-mode) | 0.3 | 0.4 | 0.5 | 0.5 | 0.6 | 0.6 | 0.6 | 0.7 | 0.7 | 0.7 | 0.7 | 0.7 |
|  | 0.23-0.39 | 0.28-0.47 | 0.35-0.58 | 0.39-0.66 | 0.43-0.71 | 0.46-0.76 | 0.48-0.81 | 0.51-0.84 | 0.53-0.88 | 0.55-0.91 | 0.56-0.94 | 0.58-0.96 |
| IVSs (M-mode) | 0.6 | 0.7 | 0.8 | 0.9 | 0.9 | 1 | 1 | 1.1 | 1.1 | 1.1 | 1.2 | 1.2 |
|  | 0.32-0.6 | 0.38-0.71 | 0.45-0.85 | 0.5-0.93 | 0.54-1 | 0.57-1.06 | 0.6-1.11 | 0.62-1.15 | 0.64-1.19 | 0.66-1.23 | 0.68-1.26 | 0.69-1.29 |
| LVs (M-mode) | 0.6 | 0.6 | 0.8 | 0.8 | 0.9 | 0.9 | 1 | 1 | 1.1 | 1.1 | 1.1 | 1.1 |
|  | 0.58-1.11 | 0.69-1.31 | 0.81-1.54 | 0.89-1.69 | 0.95-1.81 | 1-1.91 | 1.05-1.99 | 1.09-2.07 | 1.12-2.13 | 1.15-2.19 | 1.18-2.25 | 1.21-2.3 |
| LVPWs (M-mode) | 0.6 | 0.7 | 0.9 | 1 | 1.1 | 1.1 | 1.2 | 1.3 | 1.3 | 1.4 | 1.4 | 1.5 |
|  | 0.34-0.55 | 0.42-0.69 | 0.52-0.86 | 0.59-0.98 | 0.65-1.07 | 0.70-1.15 | 0.74-1.22 | 0.78-1.28 | 0.81-1.33 | 0.84-1.38 | 0.87-1.43 | 0.9-1.47 |

IVSd, in­terventricular septal thickness in diastole; IVSs, interventricular septal thickness in systole; LVDs, left ventricular internal diameter in systole; LVPWd, left ventricular posterior wall thickness in diastole; LVPWs, left ventricular posterior wall thickness in systole; LA (la), left atrial diameter in diastole from long-axis view; LA (sa), left atrial diameter from short-axis view; TAPSE, tricuspid annular plane systolic excursion

**Supplement Table 5.** Body weight-based means and 95% prediction intervals of 2D and M-mode echocardiographic parameters derived from allometric scaling parameters in 334 rhesus macaques in the range of 5 months to 4 years old.

| Echo parameters | Body weight | | | | | | | | | | | |
| --- | --- | --- | --- | --- | --- | --- | --- | --- | --- | --- | --- | --- |
|  | 1 | 2 | 4 | 6 | 8 | 10 | 12 | 14 | 16 | 18 | 20 | 22 |
| LA (sa) | 1.1 | 1.3 | 1.5 | 1.7 | 1.8 | 1.9 | 2 | 2.1 | 2.1 | 2.2 | 2.3 | 2.3 |
|  | 0.88-1.27 | 1.05-1.52 | 1.25-1.81 | 1.38-2.0 | 1.48-2.15 | 1.57-2.28 | 1.64-2.38 | 1.71-2.48 | 1.77-2.56 | 1.82-2.64 | 1.87-2.71 | 1.92-2.78 |
| Ao (sa) | 0.7 | 0.9 | 1 | 1.2 | 1.3 | 1.3 | 1.4 | 1.5 | 1.5 | 1.6 | 1.6 | 1.7 |
|  | 0.6-0.84 | 0.73-1.02 | 0.88-1.23 | 0.98-1.37 | 1.07-1.49 | 1.13-1.58 | 1.19-1.66 | 1.24-1.73 | 1.29-1.79 | 1.33-1.85 | 1.37-1.91 | 1.40-1.96 |
| LA (la) | 1.1 | 1.3 | 1.7 | 1.9 | 2 | 2.2 | 2.3 | 2.4 | 2.5 | 2.6 | 2.7 | 2.8 |
|  | 0.93-1.3 | 1.14-1.59 | 1.40-1.96 | 1.58-2.21 | 1.72-2.41 | 1.84-2.57 | 1.95-2.72 | 2.04-2.84 | 2.12-2.96 | 2.2-3.07 | 2.27-3.16 | 2.33-3.25 |
| Ao (la) | 0.5 | 0.6 | 0.8 | 0.9 | 1 | 1.1 | 1.2 | 1.2 | 1.3 | 1.3 | 1.4 | 1.4 |
|  | 0.42-0.59 | 0.53-0.75 | 0.67-0.94 | 0.77-1.08 | 0.85-1.19 | 0.92-1.28 | 0.98-1.37 | 1.03-1.44 | 1.08-1.50 | 1.12-1.57 | 1.16-1.62 | 1.2-1.68 |
| IVSd (2D) | 0.31 | 0.38 | 0.45 | 0.51 | 0.55 | 0.58 | 0.61 | 0.64 | 0.66 | 0.68 | 0.7 | 0.72 |
|  | 0.24-0.41 | 0.29-0.49 | 0.35-0.59 | 0.39-0.66 | 0.42-0.72 | 0.45-0.76 | 0.47-0.8 | 0.49-0.83 | 0.51-0.86 | 0.52-0.89 | 0.54-0.92 | 0.55-0.94 |
| LVPWd (2D) | 0.35 | 0.42 | 0.5 | 0.56 | 0.6 | 0.64 | 0.67 | 0.7 | 0.72 | 0.74 | 0.77 | 0.78 |
|  | 0.28-0.44 | 0.33-0.53 | 0.4-0.63 | 0.45-0.7 | 0.48-0.76 | 0.51-0.8 | 0.53-0.84 | 0.56-0.87 | 0.58-0.91 | 0.59-0.93 | 0.61-0.96 | 0.63-0.98 |
| IVSd (M-mode) | 0.3 | 0.4 | 0.4 | 0.5 | 0.5 | 0.6 | 0.6 | 0.6 | 0.6 | 0.7 | 0.7 | 0.7 |
|  | 0.22-0.38 | 0.26-0.46 | 0.33-0.56 | 0.37-0.63 | 0.40-0.68 | 0.42-0.72 | 0.44-0.76 | 0.46-0.79 | 0.48-0.82 | 0.50-0.85 | 0.51-0.88 | 0.53-0.90 |
| LVd (M-mode) | 1.2 | 1.4 | 1.8 | 2 | 2.2 | 2.4 | 2.5 | 2.7 | 2.8 | 2.9 | 3 | 3.1 |
|  | 0.99-1.36 | 1.23-1.69 | 1.52-2.1 | 1.73-2.39 | 1.89-2.61 | 2.03-2.8 | 2.15-2.97 | 2.26-3.11 | 2.35-3.32 | 2.24-3.37 | 2.53-3.48 | 2.6-3.59 |
| LVPWd (M-mode) | 0.3 | 0.4 | 0.5 | 0.5 | 0.6 | 0.6 | 0.6 | 0.7 | 0.7 | 0.7 | 0.7 | 0.7 |
|  | 0.24-0.39 | 0.29-0.48 | 0.35-0.58 | 0.40-0.65 | 0.43-0.71 | 0.46-0.76 | 0.49-0.8 | 0.51-0.84 | 0.53-0.87 | 0.55-0.9 | 0.56-0.93 | 0.58-0.95 |
| IVSs (M-mode) | 0.6 | 0.7 | 0.8 | 0.9 | 1 | 1 | 1.1 | 1.1 | 1.1 | 1.2 | 1.2 | 1.2 |
|  | 0.32-0.56 | 0.39-0.67 | 0.47-0.81 | 0.52-0.9 | 0.56-0.97 | 0.59-1.03 | 0.62-1.08 | 0.65-1.13 | 0.67-1.17 | 0.69-1.2 | 0.71-1.24 | 0.73-1.27 |
| LVs (M-mode) | 0.6 | 0.7 | 0.9 | 1 | 1.1 | 1.2 | 1.3 | 1.3 | 1.4 | 1.4 | 1.5 | 1.5 |
|  | 0.54-0.90 | 0.68-1.13 | 0.85-1.42 | 0.97-1.63 | 1.07-1.79 | 1.15-1.93 | 1.22-2.05 | 1.29-2.15 | 1.35-2.25 | 1.4-2.34 | 1.45-2.42 | 1.49-2.5 |
| LVPWs (M-mode) | 0.6 | 0.7 | 0.8 | 0.9 | 1 | 1.1 | 1.1 | 1.2 | 1.2 | 1.3 | 1.3 | 1.3 |
|  | 0.35-0.57 | 0.43-0.69 | 0.53-0.85 | 0.59-0.95 | 0.64-1.03 | 0.69-1.1 | 0.73-1.16 | 0.76-1.22 | 0.79-1.26 | 0.82-1.31 | 0.84-1.35 | 0.86-1.38 |

IVSd, in­terventricular septal thickness in diastole; IVSs, interventricular septal thickness in systole; LVDs, left ventricular internal diameter in systole; LVPWd, left ventricular posterior wall thickness in diastole; LVPWs, left ventricular posterior wall thickness in systole; LA (la), left atrial diameter in diastole from long-axis view; LA (sa), left atrial diameter from short-axis view; TAPSE, tricuspid annular plane systolic excursion

**Supplement Table 6.** Body weight-based means and 95% prediction intervals of 2D and M-mode echocardiographic parameters derived from allometric scaling parameters in 264 rhesus macaques in the range of 5 years to 9 years old.

| Echo parameters | Body weight | | | | | | | | | | | |
| --- | --- | --- | --- | --- | --- | --- | --- | --- | --- | --- | --- | --- |
|  | 1 | 2 | 4 | 6 | 8 | 10 | 12 | 14 | 16 | 18 | 20 | 22 |
| LA (sa) | 1.05 | 1.26 | 1.52 | 1.69 | 1.82 | 1.93 | 2.02 | 2.11 | 2.18 | 2.25 | 2.31 | 2.37 |
|  | 0.88-1.27 | 1.05-1.52 | 1.26-1.82 | 1.4-2.03 | 1.51-2.19 | 1.6-2.32 | 1.68-2.43 | 1.75-2.53 | 1.81-2.62 | 1.87-2.7 | 1.92-2.78 | 1.97-2.85 |
| Ao (sa) | 0.83 | 0.96 | 1.12 | 1.22 | 1.29 | 1.36 | 1.41 | 1.46 | 1.5 | 1.54 | 1.58 | 1.61 |
|  | 0.69-0.99 | 0.81-1.15 | 0.94-1.33 | 1.02-1.45 | 1.09-1.54 | 1.14-1.62 | 1.18-1.68 | 1.22-1.74 | 1.26-1.79 | 1.29-1.84 | 1.32-1.88 | 1.35-1.92 |
| LA (la) | 1.32 | 1.53 | 1.76 | 1.92 | 2.04 | 2.13 | 2.22 | 2.29 | 2.35 | 2.41 | 2.46 | 2.51 |
|  | 1.09-1.61 | 1.26-1.85 | 1.46-2.14 | 1.58-2.33 | 1.68-2.47 | 1.76-2.59 | 1.83-2.69 | 1.89-2.77 | 1.94-2.85 | 1.99-2.92 | 2.03-2.99 | 2.07-3.04 |
| Ao (la) | 0.65 | 0.75 | 0.87 | 0.95 | 1.02 | 1.07 | 1.11 | 1.15 | 1.18 | 1.21 | 1.24 | 1.27 |
|  | 0.54-0.78 | 0.62-0.91 | 0.73-1.05 | 0.79-1.15 | 0.84-1.22 | 0.89-1.28 | 0.92-1.34 | 0.95-1.38 | 0.98-1.42 | 1.01-1.46 | 1.03-1.49 | 1.05-1.52 |
| IVSd (2D) | 0.32 | 0.38 | 0.45 | 0.5 | 0.54 | 0.57 | 0.6 | 0.63 | 0.65 | 0.67 | 0.69 | 0.7 |
|  | 0.25-0.4 | 0.3-0.47 | 0.36-0.57 | 0.4-0.63 | 0.43-0.68 | 0.46-0.72 | 0.48-0.75 | 0.5-0.79 | 0.52-0.81 | 0.53-0.84 | 0.55-0.86 | 0.56-0.88 |
| LVPWd (2D) | 0.37 | 0.44 | 0.51 | 0.56 | 0.6 | 0.63 | 0.66 | 0.68 | 0.71 | 0.72 | 0.74 | 0.76 |
|  | 0.31-0.45 | 0.36-0.52 | 0.43-0.61 | 0.47-0.68 | 0.5-0.72 | 0.53-0.76 | 0.55-0.79 | 0.57-0.82 | 0.59-0.85 | 0.6-0.87 | 0.62-0.89 | 0.63-0.91 |
| IVSd (M-mode) | 0.3 | 0.36 | 0.43 | 0.47 | 0.5 | 0.53 | 0.56 | 0.58 | 0.6 | 0.62 | 0.63 | 0.65 |
|  | 0.23-0.4 | 0.27-0.47 | 0.32-0.56 | 0.36-0.62 | 0.38-0.67 | 0.4-0.71 | 0.42-0.74 | 0.44-0.77 | 0.45-0.79 | 0.47-0.82 | 0.48-0.84 | 0.49-0.86 |
| LVd (M-mode) | 1.38 | 1.63 | 1.91 | 2.1 | 2.25 | 2.37 | 2.47 | 2.56 | 2.64 | 2.71 | 2.78 | 2.84 |
|  | 1.16-1.65 | 1.36-1.94 | 1.6-2.28 | 1.76-2.51 | 1.88-2.68 | 1.98-2.82 | 2.07-2.95 | 2.14-3.05 | 2.21-3.15 | 2.27-3.24 | 2.33-3.32 | 2.38-3.39 |
| LVPWd (M-mode) | 0.31 | 0.38 | 0.46 | 0.51 | 0.55 | 0.59 | 0.62 | 0.65 | 0.67 | 0.69 | 0.72 | 0.73 |
|  | 0.25-0.39 | 0.3-0.47 | 0.37-0.57 | 0.41-0.64 | 0.44-0.69 | 0.47-0.74 | 0.5-0.78 | 0.52-0.81 | 0.54-0.84 | 0.56-0.87 | 0.57-0.89 | 0.59-0.92 |
| IVSs (M-mode) | 0.55 | 0.65 | 0.76 | 0.83 | 0.89 | 0.94 | 0.98 | 1.01 | 1.04 | 1.07 | 1.1 | 1.12 |
|  | 0.35-0.61 | 0.41-0.72 | 0.48-0.84 | 0.53-0.92 | 0.57-0.99 | 0.6-1.04 | 0.62-1.08 | 0.65-1.12 | 0.67-1.16 | 0.68-1.19 | 0.7-1.22 | 0.72-1.25 |
| LVs (M-mode) | 0.55 | 0.63 | 0.73 | 0.8 | 0.85 | 0.89 | 0.92 | 0.95 | 0.98 | 1 | 1.02 | 1.04 |
|  | 0.64-1.23 | 0.74-1.42 | 0.86-1.64 | 0.93-1.78 | 0.99-1.89 | 1.04-1.98 | 1.08-2.06 | 1.11-2.13 | 1.14-2.19 | 1.17-2.24 | 1.2-2.29 | 1.22-2.33 |
| LVPWs (M-mode) | 0.55 | 0.69 | 0.86 | 0.98 | 1.08 | 1.16 | 1.23 | 1.3 | 1.35 | 1.41 | 1.46 | 1.5 |
|  | 0.33-0.53 | 0.42-0.66 | 0.52-0.83 | 0.6-0.95 | 0.66-1.04 | 0.7-1.12 | 0.75-1.19 | 0.79-1.25 | 0.82-1.3 | 0.85-1.35 | 0.88-1.4 | 0.91-1.44 |

IVSd, in­terventricular septal thickness in diastole; IVSs, interventricular septal thickness in systole; LVDs, left ventricular internal diameter in systole; LVPWd, left ventricular posterior wall thickness in diastole; LVPWs, left ventricular posterior wall thickness in systole; LA (la), left atrial diameter in diastole from long-axis view; LA (sa), left atrial diameter from short-axis view; TAPSE, tricuspid annular plane systolic excursion

**Supplement Table 7.** Body weight-based means and 95% prediction intervals of 2D and M-mode echocardiographic parameters derived from allometric scaling parameters in 137 rhesus macaques in the range of 10 months to 14 years old.

| Echo parameters | Body weight | | | | | | | | | | | |
| --- | --- | --- | --- | --- | --- | --- | --- | --- | --- | --- | --- | --- |
|  | 1 | 2 | 4 | 6 | 8 | 10 | 12 | 14 | 16 | 18 | 20 | 22 |
| LA (sa) | 1.11 | 1.31 | 1.55 | 1.71 | 1.83 | 1.94 | 2.02 | 2.1 | 2.17 | 2.23 | 2.29 | 2.34 |
|  | 0.91-1.34 | 1.08-1.59 | 1.28-1.88 | 1.41-2.08 | 1.51-2.23 | 1.59-2.35 | 1.67-2.46 | 1.73-2.55 | 1,79-2.64 | 1.84-2.71 | 1.89-2.78 | 1.93-2.85 |
| Ao (sa) | 0.92 | 1.05 | 1.19 | 1.29 | 1.36 | 1.42 | 1.47 | 1.51 | 1.55 | 1.58 | 1.61 | 1.64 |
|  | 0.76-1.13 | 0.86-1.28 | 0.98-1.46 | 1.06-1.57 | 1.11-1.66 | 1.16-1.73 | 1.2-1.79 | 1.24-1.84 | 1.27-1.89 | 1.3-1.93 | 1.32-1.97 | 1.35-2.01 |
| LA (la) | 1.14 | 1.37 | 1.64 | 1.83 | 1.97 | 2.09 | 2.2 | 2.29 | 2.37 | 2.45 | 2.51 | 2.58 |
|  | 0.89-1.45 | 1.07-1.74 | 1.29-2.09 | 1.43-2.33 | 1.55-2.52 | 1.64-2.67 | 1.72-2.8 | 1.79-2.92 | 1.86-3.02 | 1.92-3.12 | 1.97-3.21 | 2.02-3.29 |
| Ao (la) | 0.67 | 0.78 | 0.9 | 0.98 | 1.04 | 1.09 | 1.13 | 1.17 | 1.2 | 1.23 | 1.26 | 1.29 |
|  | 0.54-0.82 | 0.63-0.95 | 0.73-1.1 | 0.79-1.2 | 0.84-1.28 | 0.88-1.34 | 0.92-1.39 | 0.95-1.44 | 0.98-1.48 | 1-1.52 | 1.02-1.55 | 1.04-1.58 |
| IVSd (2D) | 0.35 | 0.4 | 0.47 | 0.51 | 0.54 | 0.57 | 0.59 | 0.61 | 0.63 | 0.64 | 0.66 | 0.67 |
|  | 0.27-0.45 | 0.31-0.52 | 0.36-0.6 | 0.39-0.66 | 0.42-0.7 | 0.44-0.73 | 0.46-0.76 | 0.47-0.79 | 0.48-0.81 | 0.5-0.81 | 0.51-0.85 | 0.52-0.87 |
| LVPWd (2D) | 0.33 | 0.41 | 0.49 | 0.55 | 0.6 | 0.63 | 0.67 | 0.7 | 0.72 | 0.75 | 0.77 | 0.79 |
|  | 0.26-0.43 | 0.32-0.52 | 0.39-0.63 | 0.43-0.7 | 0.47-0.76 | 0.5-0.81 | 0.53-0.85 | 0.55-0.88 | 0.57-0.92 | 0.59-0.95 | 0.6-0.97 | 0.62-1.0 |
| IVSd (M-mode) | 0.3 | 0.36 | 0.42 | 0.47 | 0.5 | 0.53 | 0.56 | 0.58 | 0.6 | 0.62 | 0.63 | 0.65 |
|  | 0.22-0.41 | 0.26-0.49 | 0.31-0.58 | 0.34-0.64 | 0.37-0.69 | 0.39-0.73 | 0.41-0.76 | 0.42-0.79 | 0.44-0.82 | 0.45-0.85 | 0.46-0.87 | 0.47-0.89 |
| LVd (M-mode) | 1.37 | 1.6 | 1.87 | 2.05 | 2.19 | 2.31 | 2.4 | 2.49 | 2.57 | 2.64 | 2.7 | 2.76 |
|  | 1.11-1.69 | 1.3-1.98 | 1.52-2.31 | 1.66-2.54 | 1.78-2.71 | 1.87-2.85 | 1.95-2.97 | 2.02-3.07 | 2.08-3.17 | 2.14-3.25 | 2.19-3.33 | 2.23-3.41 |
| LVPWd (M-mode) | 0.35 | 0.41 | 0.48 | 0.53 | 0.57 | 0.6 | 0.63 | 0.65 | 0.67 | 0.69 | 0.71 | 0.73 |
|  | 0.27-0.46 | 0.31-0.54 | 0.37-0.64 | 0.41-0.7 | 0.44-0.75 | 0.46-0.79 | 0.48-0.83 | 0.50-0.86 | 0.51-0.88 | 0.53-0.91 | 0.54-0.93 | 0.55-0.95 |
| IVSs (M-mode) | 0.55 | 0.63 | 0.73 | 0.79 | 0.84 | 0.88 | 0.91 | 0.94 | 0.96 | 0.99 | 1.01 | 1.03 |
|  | 0.36-0.68 | 0.41-0.79 | 0.47-0.91 | 0.51-0.98 | 0.54-1.04 | 0.57-1.09 | 0.59-1.13 | 0.61-1.17 | 0.62-1.2 | 0.64-1.23 | 0.65-1.25 | 0.67-1.28 |
| LVs (M-mode) | 0.55 | 0.67 | 0.81 | 0.91 | 0.99 | 1.05 | 1.11 | 1.16 | 1.2 | 1.24 | 1.28 | 1.32 |
|  | 0.5-1.01 | 0.61-1.22 | 0.74-1.49 | 0.83-1.67 | 0.9-1.81 | 0.96-1.92 | 1.01-2.03 | 1.05-2.12 | 1.09-2.2 | 1.13-2.27 | 1.17-2.34 | 1.2-2.4 |
| LVPWs (M-mode) | 0.55 | 0.63 | 0.71 | 0.77 | 0.81 | 0.85 | 0.88 | 0.9 | 0.92 | 0.94 | 0.96 | 0.98 |
|  | 0.45-0.79 | 0.51-0.89 | 0.58-1.02 | 0.62-1.1 | 0.66-1.16 | 0.69-1.21 | 0.71-1.25 | 0.73-1.29 | 0.75-1.32 | 0.77-1.35 | 0.78-1.37 | 0.79-1.4 |

IVSd, in­terventricular septal thickness in diastole; IVSs, interventricular septal thickness in systole; LVDs, left ventricular internal diameter in systole; LVPWd, left ventricular posterior wall thickness in diastole; LVPWs, left ventricular posterior wall thickness in systole; LA (la), left atrial diameter in diastole from long-axis view; LA (sa), left atrial diameter from short-axis view; TAPSE, tricuspid annular plane systolic excursion

**Supplement Table 8.** Body weight-based means and 95% prediction intervals of 2D and M-mode echocardiographic parameters derived from allometric scaling parameters in 94 rhesus macaques over 15 years old.

| Echo parameters | Body weight | | | | | | | | | | | |
| --- | --- | --- | --- | --- | --- | --- | --- | --- | --- | --- | --- | --- |
|  | 1 | 2 | 4 | 6 | 8 | 10 | 12 | 14 | 16 | 18 | 20 | 22 |
| LA (sa) | 1.31 | 1.46 | 1.62 | 1.72 | 1.79 | 1.85 | 1.9 | 1.95 | 1.99 | 2.02 | 2.06 | 2.09 |
|  | 1.07-1.61 | 1.18-1.79 | 1.31-1.99 | 1.4-2.11 | 1.46-2.21 | 1.51-2.28 | 1.55-2.34 | 1.58-2.4 | 1.62-2.45 | 1.65-2.49 | 1.67-2.53 | 1.7-2.57 |
| Ao (sa) | 0.99 | 1.11 | 1.24 | 1.32 | 1.38 | 1.43 | 1.48 | 1.51 | 1.55 | 1.58 | 1.6 | 1.63 |
|  | 0.8-1.22 | 0.9-1.36 | 1-1.52 | 1.07-1.62 | 1.12-1.7 | 1.16-1.76 | 1.2-1.82 | 1.23-1.86 | 1.26-1.9 | 1.28-1.94 | 1.3-1.97 | 1.32-2 |
| LA (la) | 1.31 | 1.5 | 1.73 | 1.87 | 1.98 | 2.07 | 2.15 | 2.21 | 2.27 | 2.33 | 2.38 | 2.42 |
|  | 1.07-1.6 | 1.23-1.84 | 1.41-2.11 | 1.53-2.29 | 1.62-2.43 | 1.69-2.54 | 1.75-2.63 | 1.81-2.71 | 1.86-2.78 | 1.9-2.85 | 1.94-2.91 | 1.98-2.97 |
| Ao (la) | 0.53 | 0.67 | 0.83 | 0.95 | 1.04 | 1.11 | 1.18 | 1.24 | 1.29 | 1.34 | 1.39 | 1.43 |
|  | 0.42-0.68 | 0.52-0.85 | 0.65-1.06 | 0.74-1.2 | 0.82-1.32 | 0.88-1.42 | 0.93-1.5 | 0.98-1.58 | 1.02-1.65 | 1.06-1.71 | 1.09-1.77 | 1.13-1.82 |
| IVSd (2D) | 0.57 | 0.58 | 0.6 | 0.61 | 0.62 | 0.62 | 0.63 | 0.63 | 0.64 | 0.64 | 0.64 | 0.64 |
|  | 0.43-0.76 | 0.44-0.78 | 0.45-0.8 | 0.46-0.81 | 0.46-0.82 | 0.47-0.83 | 0.47-0.84 | 0.47-0.84 | 0.48-0.85 | 0.48-0.85 | 0.48-0.85 | 0.48-0.86 |
| LVPWd (2D) | 0.52 | 0.56 | 0.61 | 0.63 | 0.65 | 0.67 | 0.68 | 0.69 | 0.7 | 0.71 | 0.72 | 0.72 |
|  | 0.43-0.64 | 0.46-0.68 | 0.5-0.74 | 0.52-0.77 | 0.54-0.79 | 0.55-0.81 | 0.56-0.83 | 0.57-0.84 | 0.58-0.85 | 0.58-0.86 | 0.59-0.87 | 0.6-0.88 |
| IVSd (M-mode) | 0.44 | 0.47 | 0.5 | 0.52 | 0.53 | 0.54 | 0.55 | 0.56 | 0.56 | 0.57 | 0.58 | 0.58 |
|  | 0.32-0.61 | 0.34-0.65 | 0.36-0.69 | 0.38-0.71 | 0.38-0.73 | 0.39-0.75 | 0.4-0.76 | 0.4-0.77 | 0.41-0.78 | 0.41-0.79 | 0.42-0.8 | 0.42-0.8 |
| LVd (M-mode) | 1.49 | 1.66 | 1.86 | 1.98 | 2.08 | 2.15 | 2.22 | 2.27 | 2.32 | 2.37 | 2.41 | 2.44 |
|  | 1.2-1.84 | 1.34-2.06 | 1.5-2.31 | 1.6-2.46 | 1.67-2.58 | 1.73-2.67 | 1.79-2.75 | 1.83-2.82 | 1.87-2.88 | 1.91-2.94 | 1.94-2.99 | 1.97-3.03 |
| LVPWd (M-mode) | 0.38 | 0.44 | 0.51 | 0.55 | 0.59 | 0.62 | 0.64 | 0.66 | 0.68 | 0.7 | 0.72 | 0.73 |
|  | 0.3-0.48 | 0.35-0.55 | 0.41-0.64 | 0.44-0.7 | 0.47-0.74 | 0.49-0.78 | 0.51-1.24 | 0.53-0.83 | 0.54-0.86 | 0.56-0.88 | 0.57-0.9 | 0.58-0.92 |
| IVSs (M-mode) | 0.55 | 0.61 | 0.67 | 0.72 | 0.75 | 0.77 | 0.79 | 0.81 | 0.83 | 0.84 | 0.85 | 0.87 |
|  | 0.43-0.79 | 0.48-0.88 | 0.53-0.97 | 0.56-1.03 | 0.58-1.07 | 0.6-1.11 | 0.62-1.14 | 0.63-1.16 | 0.65-1.19 | 0.66-1.21 | 0.67-1.23 | 0.68-1.24 |
| LVs (M-mode) | 0.55 | 0.64 | 0.74 | 0.8 | 0.85 | 0.9 | 0.93 | 0.96 | 0.99 | 1.02 | 1.04 | 1.06 |
|  | 0.51-1.1 | 0.59-1.27 | 0.68-1.47 | 0.74-1.61 | 0.79-1.71 | 0.83-1.79 | 0.86-1.86 | 0.89-1.92 | 0.92-1.98 | 0.94-2.03 | 0.96-2.07 | 0.98-2.12 |
| LVPWs (M-mode) | 0.55 | 0.67 | 0.82 | 0.93 | 1.01 | 1.08 | 1.14 | 1.19 | 1.24 | 1.28 | 1.32 | 1.36 |
|  | 0.37-0.63 | 0.45-0.77 | 0.55-0.94 | 0.62-1.06 | 0.67-1.15 | 0.72-1.23 | 0.76-1.29 | 0.79-1.35 | 0.82-1.41 | 0.85-1.46 | 0.88-1.5 | 0.9-1.54 |

IVSd, in­terventricular septal thickness in diastole; IVSs, interventricular septal thickness in systole; LVDs, left ventricular internal diameter in systole; LVPWd, left ventricular posterior wall thickness in diastole; LVPWs, left ventricular posterior wall thickness in systole; LA (la), left atrial diameter in diastole from long-axis view; LA (sa), left atrial diameter from short-axis view; TAPSE, tricuspid annular plane systolic excursion
